# Omics Analyses of *Trichomonas vaginalis* Actin and Tubulin and Their Participation in Intercellular Interactions and Cytokinesis

**DOI:** 10.3390/genes13061067

**Published:** 2022-06-15

**Authors:** Sebastián Lorenzo-Benito, Luis Alberto Rivera-Rivas, Lizbeth Sánchez-Ayala, Jaime Ortega-López, Octavio Montes-Flores, Daniel Talamás-Lara, Rossana Arroyo

**Affiliations:** 1 Departamento de Infectómica y Patogénesis Molecular, Centro de Investigación y de Estudios Avanzados del Instituto Politécnico Nacional (CINVESTAV-IPN), Av. IPN #2508, Col. San Pedro Zacatenco, Alcaldía Gustavo A. Madero, Mexico City CP 07360, Mexico; sebastian.lorenzo@cinvestav.mx (S.L.-B.); larr_fremx@hotmail.com (L.A.R.-R.); lizbeth.sanchez@cinvestav.mx (L.S.-A.); dtalamas@cinvestav.mx (D.T.-L.); 2 Departamento de Biotecnología y Bioingeniería, CINVESTAV-IPN. Av. IPN #2508, Col. San Pedro Zacatenco, Alcaldía Gustavo A. Madero, Mexico City CP 07360, Mexico; jortega@cinvestav.mx (J.O.-L.); red_bull133@hotmail.com (O.M.-F.)

**Keywords:** actin, amoeboid, cyst-like structure, cytokinesis, intercellular interactions, nanotube-like structures (NTS), ovoid, paradesmose, *Trichomonas vaginalis*, tubulin

## Abstract

Actin and tubulin proteins from *Trichomonas vaginalis* are crucial for morphogenesis and mitosis. This parasite has 10 and 11 genes coding bonafide actin and tubulin proteins, respectively. Hence, the goal of this work was to analyze these actin and tubulin genes, their expression at the mRNA and protein levels, and their parasite localization in intercellular interaction and cytokinesis. Representative bonafide actin (*tvact1*) and tubulin (*tvtubα1*) genes were cloned into and expressed in *Escherichia coli*. The recombinant proteins TvACT1r and TvTUBα1r were affinity purified and used as antigens to produce polyclonal antibodies. These antibodies were used in 1DE and 2DE WB and indirect immunofluorescence assays (IFA). By IFA, actin was detected as a ring on the periphery of ameboid, ovoid, and cold-induced cyst-like parasites, on pseudopods of amoeboid parasites, and in cytoplasmic extensions (filopodia) in cell–cell interactions. Tubulin was detected in the axostyle, flagellum, undulating membrane, and paradesmose during mitosis. Paradesmose was observed by IFA mainly during cytokinesis. By scanning electron microscopy, a tubulin-containing nanotubular structure similar to the tunneling nanotubes (TNTs) was also detected in the last stage of cytokinesis. In conclusion, actin and tubulin are multigene families differentially expressed that play important roles in intercellular interactions and cytokinesis.

## 1. Introduction

*Trichomonas vaginalis* (*Tv*) is the protozoan parasite that causes human trichomoniasis, which is a sexually transmitted infection with a worldwide distribution. Approximately 142 million new cases of trichomoniasis are registered every year in people between 15 and 49 years old [1]. *Tv* infection increases the risk of acquiring the human immunodeficiency virus, developing cervical and prostate cancer, and pregnancy distress effects, such as preterm delivery [2,3,4,5]. This parasite has three morphologies: an ovoid or piriform freely swimming form, an amoeboid form attached to vaginal epithelial cells or inert surfaces, and a cyst-like form when grown in the absence of nutrients or exposed to cold. The latter form has been isolated from cervical neoplasia patients and is capable of binding to epithelial cells [6,7,8,9]. 

*Tv* has a very dynamic cytoskeleton that allows rapid changes in morphology, depending on environmental conditions. Actin and tubulin proteins and their associated proteins participate in this process. The *Tv* genome encodes a variety of canonical actin-associated, tubulin-associated, and regulating proteins, such as α-actinins, capZ, cofilin, formin, and the Arp2/3 complex, as well as fimbrin, which is an actin polymerizing-protein, and proteins of the EB family, IFT, MAPs, and dynein [10,11]. Several *Tv* proteins belong to multigene families, among which actin and tubulin are not exceptions [10,11]. Actin genes have been used to typify *Tv* strains, and eight genotypes have been found worldwide [12,13,14]. Although this parasite has 29 DNA segments coding for putative actin and 31 coding for putative tubulin, proteomic and mass spectrometry analyses showed that only 20 actin-like and 24 tubulin-like protein fragments have been reported in the different proteomes. Moreover, these gene segments are differentially expressed among strains and culture conditions [15,16,17,18,19,20,21,22,23,24,25,26,27,28,29,30]. By indirect immunofluorescence assays (IFAs) using a heterologous anti-actin monoclonal antibody (mAb) and phalloidin, F-actin was detected diffused in the cytoplasm of both flagellated and amoeboid forms. By 2DE WB also using heterologous antibodies, four actin spots were detected, two of which showed the greatest intensity [31]. By IFAs, tubulin presence has been detected in the axostyle, flagella, costa, pelta, basal body, and paradesmosa. Moreover, tubulin posttranslational modifications (PTMs), such as acetylation and polyglutamylation, have been reported [32,33,34,35,36,37,38,39,40].

The actin and tubulin cytoskeleton in *Tv* is crucial for its virulence, morphological transformation, and mitosis. Actin is important for *Tv* attachment to host cells to establish infection (cytoadherence) and morphological transformation, through the formation of filopodia and pseudopodia [6]. Tubulin also contributes to parasite motility and host–cell interactions [41]. Data suggest that actin and tubulin could participate in parasite–parasite communication, through membrane-mediated intercellular bridges, such as tunneling nanotubes (TNT’s) that contain cytoskeletal components, such as actin and tubulin [42,43,44,45]. Thus, the goal of this work was to analyze data from the *Tv* genome sequence database including all the gene segments encoding actin-like and tubulin-like domains and expressed sequence tags (ESTs) and reported proteomes of actin and tubulin multigene families. Additionally, we also focused on the localization of bonafide TvACT and TvTUB proteins in different *Tv* morphologies, in cell–cell interactions, and during *Tv* cytokinesis using generated homologous antibodies against selected recombinant proteins.

## 2. Materials and Methods

### 2.1. Cell Culture

The CNDC147 *Tv* Mexican fresh clinical isolate was grown in trypticase-yeast extract-maltose (TYM) Diamond medium supplemented with 10% heat-inactivated adult bovine serum for 24 h at 37 °C and maintained for up to two weeks in in vitro culture by daily passages. 

### 2.2. Cloning and Expression of the Recombinant Actin Protein

The complete actin (1131 bp; TVAG_160060) and tubulin (1359 bp; TVAG_359090) genes were amplified by PCR using genomic DNA from the CNCD147 *Tv* isolate as a template and a proof-reading polymerase. For *tvact1*, a sense primer (5′-GAGCTTGGATCCATGGCTGAAGAAGACGTTCAG-3′) and an antisense primer (5′-GAGCTTAAGCTTTTAGAAGCACTTGCGGTGAAC-3′) were used. For *tvtubα1*, a sense primer (5′-GAGCTTCATATGCGTGAAGTTATTTCTATC-3′), and an antisense primer (5′-GAGCTTGGATCCTTACATTTCGCCACCATCTTC-3′) were used. Each primer pair contained the restriction sites *Bam*HI/*Hind*III and *Nde*I/*Bam*HI. The amplicons were cloned into the pGEM-T easy vector for sequencing. The same sense primer and a new antisense primer that included a *Pst*I restriction site (5′-GAGCTTCTGCAGTTAGAAGCACTTGCGGTGAAC-3′) were used for subcloning the *tvact1* gene into the pCold I prokaryotic expression vector with a polyhistidine tag (Takara Bio Inc., Mountain View, CA, USA). The *tvtubα1* gene was subcloned into the pCri1b expression vector [46] with a polyhistidine tag and an MBP (Maltose Binding Protein) Tag of ~42 kDa molecular weight. Recombinant protein expression was performed in the *Escherichia coli* BL21 (DE3) strain, induced with 1 mM isopropyl-β-D-1-thiogalactopyranosides for 16 h at 16 °C for actin and 37 °C for tubulin, and analyzed by denatured polyacrylamide gel electrophoresis (SDS-PAGE). Recombinant TvACT1 and TvTUBα1 proteins were purified by affinity chromatography using Ni-Sepharose 6 Fast Flow columns (GE Healthcare-Amersham Biosciences, Chalfont St Giles, Hatfield, UK), as recommended by the manufacturer. Purified TvACT1r and TvTUBα1 were used as antigens for antibody production.

### 2.3. Generation of Polyclonal Anti-TvACT1r and Anti-TvTUBα1r Antibodies

The rabbit anti-TvACT1r polyclonal antibody was produced by intramuscularly inoculating a four-week-old male New Zealand rabbit twice at three-week intervals with 300 µg of the purified TvACT1r protein. The mouse anti-TvTUBα1r polyclonal antibody was produced by subcutaneously inoculating 15 female BALB/c mice twice with a two-week interval with 50 µg of the purified TvTUBα1r protein per mouse. In both cases, TiterMax^®^ Gold Adjuvant (Sigma-Aldrich, St Louis, MO, USA) was used for each immunization. The anti-TvACT1r and anti-TvTUBα1r sera were obtained 15 days after the last immunization and titrated by WB using nitrocellulose (NC) membranes containing total trichomonad protein extracts. The anti-TvACT1r and anti-TvTUBα1r antibodies were used in WB assays and IFAs. Secondary antibodies were used as negative controls in all experiments with antibodies.

### 2.4. Protein Preparation and SDS–PAGE in 1DE and 2DE

Total trichomonad protein extracts were obtained from 2 × 10^7^ parasites by 10% trichloroacetic acid (TCA) precipitation at 4 °C overnight [47]. The protein pellet was resuspended in sample buffer, boiled for 5 min, and analyzed by 1D SDS-PAGE (1DE) on 12% polyacrylamide gels. For 2DE, we used TCA-precipitated proteins from 1 × 10^7^ parasites. The protein pellet was washed with PBS and cold acetone, air-dried, resuspended in 150 µL Rehydration Buffer (Bio-Rad Laboratories, Hercules, CA, USA), centrifuged, and the supernatant was loaded into 7 cm ReadyStrip IPG strips (linear pH gradient 4–7; Bio-Rad) and actively rehydrated for 16 h at 20 °C and 50 V. Protein isoelectric focusing (IEF) was performed using a protean IEF cell (Bio-Rad) in four steps: 250 V for 20 min, 4000 V for 3 h, a gradual increase from 4000 V to 10,000 V-h and holding at 500 V. After IEF, the IPG strips were equilibrated for reduction and alkylation in equilibration buffer (Bio-Rad). For the second dimension, the proteins were resolved by SDS-PAGE on 12% polyacrylamide gels. The protein gels were Coomassie Brilliant Blue (CBB) stained or transferred onto NC membranes (Bio-Rad) for WB detection.

### 2.5. MS Analysis by LC-ESI–HDMS

From the 2DE gel, the spots corresponding to actin and tubulin (spots 1, 2, 3 and 1, 2, 3, 4, respectively) were sliced and enzymatically digested with trypsin according to the modified protocol of Schevenko [48]. Afterwards, the digested peptides were loaded and separated on an HSS T3 C18 Column (Waters, Milford, MA, USA) measuring 75 µm × 150 mm with a 100 Å pore size and 1.8 µm particle size; using an UPLC ACQUITY M-Class instrument (Waters) with similar mobile phases according to the following gradient: 0 min 7% B, 30.37 min 40% B, 32.03–35.34 min 85% B, and 37–47 min 7% B at a flow rate of 400 nL/min and 45 °C. Spectral data were acquired using a Synapt G2-Si mass spectrometer with electrospray ionization and ion mobility separation (Waters) with a data-independent acquisition approach in HDMS^E^ (Waters). The tuning page for the ionization source was set up with the following parameters: 2.75 kV in the sampler capillary, 30 V in the sampling cone, 30 V in the source offset, 70 °C for the source temperature, 0.5 bar for the nanoflow gas, and 120 L/h for the purge gas flow rate. Two chromatograms were acquired (low- and high-energy chromatograms) in positive mode in the 50–2000 *m*/*z* range with a scan time of 500 ms. No collision energy was applied to obtain the low-energy chromatogram. However, in the case of the high-energy chromatograms, the precursor ions were fragmented in the transfer cell using a collision energy ramp of 19–55 V. The generated raw files containing MS and tandem mass spectra were deconvoluted and compared against the *Tv* genome (https://trichdb.org/trichdb/app accessed on 9 May 2021) using ProteinLynx Global SERVER v3.0.3 (Waters). The workflow parameters were trypsin as a cut enzyme and one missed cleavage allowed; carbamidomethyl (C) as a fixed modification; and amidation (N-term), deamidation (N, Q), oxidation (M), phosphorylation (S, T, Y) as variable modifications. Automatic peptide and fragment tolerance, a minimum fragment ion matches per peptide of 2, a minimum fragment ion matches per protein of 5, a minimum peptide matches per protein of 1, and a false discovery rate ≤4% were used. All identifications had ≥95% reliability (Protein AutoCurate green).

### 2.6. Western Blot (WB) Assays

The WB assays were performed using TCA-precipitated proteins separated by SDS-PAGE on 12% polyacrylamide gels, which were transferred onto NC membranes (0.2 µm pore size; Bio-Rad) and blocked with 5% nonfat dried milk in PBS-0.1% Tween 20 (PBS-T20). The NC membranes were incubated for 1 h at 37 °C with the following primary antibodies diluted in PBS-T20: anti-TvACT1r rabbit antibody (1:7000 dilution), anti- TvTUBα1r mouse polyclonal antibody (1:15,000 dilution), and anti-α-tubulin mouse monoclonal antibody (1:1000 dilution; Invitrogen, #13-800). After ten washes with PBS-T20, the NC membranes were incubated for 1 h at 37 °C with a horseradish peroxidase-conjugated goat anti-rabbit or anti-mouse IgG secondary antibody (1:9000). The NC membranes were developed by a color reaction for anti-actin and anti-tubulin homolog polyclonal antibodies with 4-chloro-naphthol as a substrate or by a chemiluminescence system (SuperSignal West Pico Chemiluminescent Substrate, Thermo Scientific-Pierce, Rockford, IL, USA) for the anti-tubulin heterologous monoclonal antibody. Images were captured in a ChemiDoc XRS System (Bio-Rad).

### 2.7. Indirect Immunofluorescence Assay (IFA)

To determine the cellular localization of actin and tubulin by IFA, parasites grown for 24 h at 37 °C were used. The parasites were washed three times with cold PBS and fixed (ovoid) or incubated for 6 h on ice (cyst-like) or were transferred to fresh medium and incubated for 2 h at 37 °C on coverslips (ameboid). The parasites were fixed with 4% paraformaldehyde for 30 min at 37 °C and permeabilized with 0.5% Triton X-100 for 10 min at room temperature (RT). For colocalization assays, the parasites were incubated with rabbit polyclonal anti-TvACT1r (1:500 dilution) and mouse monoclonal heterologous anti-tubulin (1:100 dilution) antibodies overnight at 4 °C. Then, the parasites were incubated with a fluorescein isothiocyanate (FITC)-conjugated secondary anti-rabbit antibody (1:100 dilution; #65-6111, Invitrogen, Waltham, MA, USA) and with Alexa 594-conjugated secondary anti-mouse antibody (1:100 dilution; #A11005, Invitrogen for 3 h at 4 °C and mounted with Vectashield-DAPI (4′,6′-diaminide-2′-phenylindole dihydrochloride) mounting solution (Vector Laboratories, Burlingame, CA, USA). For the anti- TvTUBα1 (1:500 dilution), anti-acetylated tubulin (anti-TUBAcet) (1:1000 dilution; #22180410, Sigma-Aldrich, St. Louis, MO, USA), and anti-polyglutamylated tubulin (anti-TUBPGlu) (1:1000 dilution; #019M4837 V, Sigma-Aldrich) antibodies, the parasites were adhered to coverslips for 30 min in fresh medium and fixed with 4% formaldehyde for 30 min at RT. In this assay, all primary antibodies were incubated for 1 h at RT followed by incubation with Alexa 647-conjugated secondary anti-mouse antibody (1:300 dilution; #A32728, Invitrogen) for 1 h at 4 °C and mounted with Vectashield-DAPI. All samples were analyzed by confocal microscopy using a Zeiss microscope and Zen 2012 software (Carl Zeiss, Jena, Germany).

### 2.8. Scanning Electron Microscopy 

After 50 min of incubation of parasites on coverslips at 37 °C with fresh medium, the samples were fixed with 2.5% (*v*/*v*) glutaraldehyde in 0.1 M sodium cacodylate buffer pH 7.2, dehydrated in increasing concentrations of ethanol, critical-point dried with liquid CO_2_ (31 °C and 1100 psi) using a Samdri 780 apparatus (Tousimis Corp., Rockville, MD, USA), and coated with gold particles in an ion sputtering device (JEOLJFC-1100). The samples were then examined with a field emission JEOL-JSM7100F scanning electron microscope (JEOL Ltd., Tokyo, Japan).

## 3. Results

### 3.1. Actin and Tubulin Genes Present in the Genome of T. vaginalis

By searching the genome sequence database of *Tv*, we found 29 and 31 DNA segments encoding actin-like and tubulin-like protein fragments, respectively, in different contigs. For actin-like protein fragments, the DNA segment sizes ranged from 369 to 1131 nucleotides (nt), with 24 of the 29 sequences presenting evidence of expression as ESTs. For tubulin-like protein fragments, the DNA segment sizes ranged from 147 to 1359 nt, with 24 of the 31 presenting ESTs, even though some corresponded to partial sequences (Supplementary Tables S1 and S2; Figures S1 and S2). Ten actin gene sequences (*tvact1*–*tvact10*) showed ~70% identity with the α-actin gene of *Homo sapiens* (*huact*) and ~74% at the protein level with HuACT and were considered complete genes since these presented all the regulatory elements at the 5′ and 3′ end for their transcription and polyadenylation (Supplementary Table S3). These genes were included in group I and were considered genes encoding bonafide actin from *Tv* (Supplementary Table S1). Seven actin genes (*tvact1*–*tvact7*) encoded proteins with 100% identity. Eleven tubulin gene sequences were considered complete genes that encode bonafide tubulins in *Tv* (group I), since these presented all the regulatory elements at the 5′ and 3′ end for their transcription and polyadenylation (Supplementary Tables S2 and S4). Seven showed high identity at the gene and protein level with the α-tubulin (HuTUBα) and four with a β-tubulin (HuTUBβ) from *H. sapiens* and were divided into subgroups I-A and I-B, respectively. Within each subgroup, the tubulin-coding genes at the aa level showed 100% identity (*tvtubα1*–*tvtubα5* and *tvtubα6*–*tvtubα7*) and (*tvtubβ2*–*tvtubβ4*), respectively, despite presenting certain changes at the nt level. These gene sequences encoded proteins with sizes of 376 aa for actin and 452 aa for TvTUBα and 447 aa for TvTUBβ, with molecular weights and pIs similar to those previously reported for bonafide actin and tubulin proteins [31] (Supplementary Tables S1 and S2; Figures S1 and S2). Three more actin-like DNA fragments (TVAG_512800, TVAG_150270, and TVAG_534990) showed high identity to the group I actin genes but are incomplete gene sequences that lack part of the regulatory elements, the start or stop codons, the first and/or last aa residues or even it has a track of 100 unknown nucleotides at its 3′ end, which probably indicates an error in its sequencing (Supplementary Figure S1; Tables S1 and S5). These sequences were part of group II-A. Similar results were found for the tubulin-like coding DNA fragment that was part of group II-A (Supplementary Figure S2; Tables S2 and S6). Other gene sequences homologous to actin and tubulin that are more divergent from group I were classified in subgroups II-B and II-C, respectively. These subgroups included sequences that present start and stop codons, with different ORF sizes, that may or may not have regulatory elements at their 5′ and 3′ ends. Subgroup II-B includes those with evidence of expression according to the analyzed proteomes (Supplementary Tables S7 and S8) and were called actin-like or tubulin-like protein fragments. Subgroup II-C includes those that do not have evidence of expression at the protein level and should be considered hypothetical proteins. 

In conclusion, based on the in silico analysis, group I concentrates the 10 bonafide actin- and 11 bonafide tubulin-encoding genes, which also have the highest number of ESTs (Supplementary Tables S1 and S2), suggesting that these genes were expressed in trichomonads under most culture conditions studied. 

### 3.2. In Silico Analysis of the Deduced 29 Actin-like and 31 Tubulin-like Protein Fragments Found in the Genome of T. vaginalis 

We performed an in silico analysis of the deduced 29 TvACT-like and 31 TvTUB-like protein fragments, looking for similarities and differences among them. Supplementary Figures S1 and S2 show a schematic representation of each deduced actin-like and tubulin-like protein fragment and the putative functional domains predicted by Pfam, ScanProsite, and InterProscan and the missing regions could be appreciated for some of them. The bonafide actin proteins (TvACT1 to TvACT10) present identical domains (group I). The subgroup II-A members have functional domains similar to the bonafide proteins. However, the TVAG_512800 and TVAG_534990, are incomplete proteins that lack the first 10 and 138 aa, respectively, at their N-termini. At the level of putative functional domains, all have an ATP-binding site, an actin-like ATPase domain, a putative phospholipase D-type domain, and a leucine zipper domain. Only TVAG_534990 lacks the ATP-binding site at its N-terminal end due to its partial sequence. Subgroups II-B and II-C present their particularities. The TVAG_189860 protein lacks a conserved actin domain and a putative phospholipase D-type domain, and its leucine zipper domain position is exchanged with that of the actin-like ATPase domain. The deduced aa sequences TVAG_495500, TVAG_339989, TVAG_043970, TVAG_071770, TVAG_354260, TVAG_161530, TVAG_247170, TVAG_225210, and TVAG_23810025 have a putative actin-like ATPase domain and lack an ATP-binding site, putative phospholipase D-type, and leucine zipper domains. The TVAG_434970 protein fragment lacks an ATP-binding site, putative phospholipase D-type, and leucine zipper domains. Instead, other putative domains, such as DUF3920, whose function is unknown, and an alanine ligase are present in this protein. The TVAG_371880 protein fragment has an ATP binding site and two putative TetR domains that are tetracycline repressor-like C-terminal domains. The TVAG_027630 protein fragment is more divergent from all other TvACT proteins. It is predicted to have two HiaBD2_N domains (a trimeric domain of the autotransporter adhesin), an oxygenase 2OGFeDO domain, and an actin-like ATPase domain. The TVAG_276270 protein fragment contains an SGS domain present in proteins that bind to calcicline, which is included in the actin-like ATPase domain. The TVAG_094140 protein fragment has a phosphopantetheine-binding site and an ATP-binding site in addition to an actin-like ATPase domain. The protein TvACT29 only presents a partial actin-like ATPase domain at the C-terminal end (Supplementary Figure S1). 

The tubulin proteins TvTUBα1-TvTUBα7, TVAG_5239808, TVAG_519620, TvTUBβ1-TvTUBβ4, TVAG_525430, TVAG_065740, TVAG_109820; TVAG_148390, TVAG_338530, and TVAG_184510 present a tubulin/Ftsz family GTPase domain, GTP-binding site, and a tubulin C-terminal domain, while TVAG_024080, TVAG_200200, TVAG_148400, TVAG_073800, TVAG_448410, TVAG_289290, TVAG_043330, and TVAG_257730 only present the tubulin C-terminal domain. The TVAG_345420, TVAG_073810, TVAG_369500, and TVAG_207590 proteins only present a tubulin/FtsZ family GTPase domain that includes a GTP-binding site. The TvTUB proteins that present only one domain are proteins truncated at the N- or C-terminus (Supplementary Figure S2). 

Moreover, we also performed a 3D structure prediction for each deduced actin-like and tubulin-like protein fragment of the *Tv* genome database obtained in the Swiss Model Server from EXPASY (https://swissmodel.expasy.org/, accessed on 26 May 2022) using the deduced aa sequence. These data were one of the parameters to consider for the identification of possible bonafide actin and tubulin proteins (group I) and actin-like and tubulin-like protein segments (group II) in *Tv* (Supplementary Tables S9 and S10). 

### 3.3. In Silico Analysis of the tvact1 and tvtubα1 Genes and TvACT1 and TvTUBα1 Proteins

**Figure 1:**
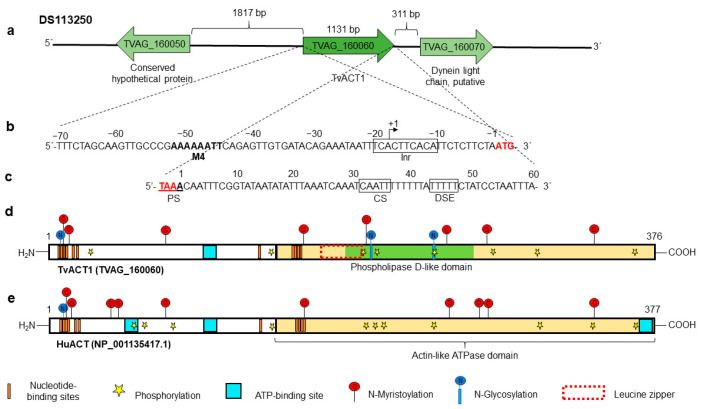
In silico analysis of the *tvact1* genomic context and the regulatory regions and PTMs of the TvACT1 and HuACT proteins. (**a**) Contig DS113250, TVAG_160060 corresponds to the *tvact1* gene (1131 nt) with intergenic regions of 1817 and 311 bp containing the other genes (TVAG_160050 and TVAG_160070, respectively) present in the same contig. (**b**) 5′ 70 nt region upstream of the start codon (**ATG**) analyzed for regulatory elements containing initiator promoter elements in tandem (Inr) (black box) and motif 4 (in bold letters). The +1 indicates the putative transcription start site. (**c**) 3′ 70 nt region downstream of the stop codon (**TAA**) analyzed for polyadenylation regulatory elements (black boxes). It contains one putative polyadenylation signal (PS) coupled to the stop codon (red letters), one putative cleavage site (CS), and a T-rich downstream element (DSE). (**d**,**e**) Schemes of the TvACT1 and HuACT proteins (TVAG_160060 and NP_001135417.1, respectively). Both proteins have an ATPase domain (cream box); ATP-binding site(s) (turquoise box), with one for TvACT1 and three for HuACT; and multiple putative nucleotide-binding sites (orange bar). TvACT1 has a putative leucine zipper (red-dotted box) and a phospholipase D-like domain (green bar). Both proteins show PTMs, such as putative sites for phosphorylation (yellow star), N-glycosylation (blue circle with vertical line), and N-myristoylation (red circle with vertical line).

Based on our previous analyses, in addition to the fact that these are sequences with greater identity with the cloned genes of the *Tv* CNCD147 isolate, we selected the *tvact1* (TVAG_160060) and *tvtubα1* (TVAG_359090) genes as reference sequences for the next set of analyses. We first performed an in silico analysis, searching for the gene regulatory regions and protein putative PTMs. The *tvact1* gene is localized at position 109535–110704 nt of the DS113250 contig flanked by a conserved hypothetical protein (TVAG_160050) and a putative dynein light chain (TVAG_160070). The 5′-intergenic region is 1817 nt long, and the 3′-intergenic region is 311 nt long (Figure 1a). The in silico analysis of 70 nt up- and downstream of the *tvact1* ORF showed putative regulatory sequences for basal transcription and mRNA polyadenylation, respectively. The 5′-intergenic region contains two putative initiator promoter elements (Inr) (the core promoter element essential for transcription [48,49]) in tandem (TCACTTCACA) located at −11 to −20 nt upstream from the translation initiation ATG codon in the *tvact1* gene. A motif 4 sequence (M4) with a consensus sequence **AAAAAATT** at −45 to −52 nt was also found (Figure 1b) [50,51]. The 3′-regulatory region of the *tvact1* gene has one putative polyadenylation site (PS), **TAAA**, which includes the stop codon and one consensus cleavage site (CS) CAATT at +32 to +36 nt downstream of the stop codon. It also has a T-rich (TTTTT) downstream element (DSE) between +44 and +48 nt (Figure 1c) [52,53].

The complete *tvact1* gene (1131 nt) encodes a 376 aa protein (TvACT1) with a predicted molecular weight of ~42 kDa and a pI of 4.82. It was compared to the human *huact* gene as a reference, which encodes a 377 aa protein (HuACT) with a molecular weight of ~42 kDa and a pI of 5.24. These proteins have 74.3% aa sequence identity. The in silico analysis showed that TvACT1 has one ATP-binding site, whereas HuACT has three (turquoise box), and a potential ATPase domain (cream box) of actin, as well as putative nucleotide-binding sites (orange bars) were also detected in both proteins (Figure 1d,e). TvACT1 and HuACT have three and one putative N-glycosylation sites, respectively. In TvACT1, these sites are located at Asp13, 202, and 240. In HuACT, the site is located at residue Asp14. The in silico analysis also predicted putative sites for N-myristoylation at residues Gly14, Gly16, Gly75, Gly159, Gly198, Gly246, Gly274, and Gly344 in TvACT1 and at positions Gly15, Gly17, Gly44, Gly50, Gly76, Gly160, Gly247, Gly270, Gly275, and Gly345 in HuACT. However, there are no reports detecting the last two types of PTMs in any actin protein. Therefore, these predicted PTMs should be taken with caution until are experimentally demonstrated. Additionally, TvACT1 presented 8 putative phosphorylation sites at Ser23, Ser147, Thr195, Thr203, Thr242, Thr278, Thr325, and Thr359, whereas HuACT presented 11 at Ser62, Ser147, Ser236, Ser340, Ser360, Thr68, Thr79, Thr196, Thr200, Thr204, and Thr326 (Figure 1d,e). Some differences were found, i.e., TvACT1 has a putative phospholipase D-like domain at the C-terminus of MIT (microtubule interacting and transport) that binds avidly to phosphoinositide-containing membranes [54], whereas HuACT does not. Another difference between TvACT1 and HuACT is that the former has a leucine zipper (red-dotted box) located between amino acids 172 and 193, and the latter does not (Figure 1d,e).

**Figure 2:**
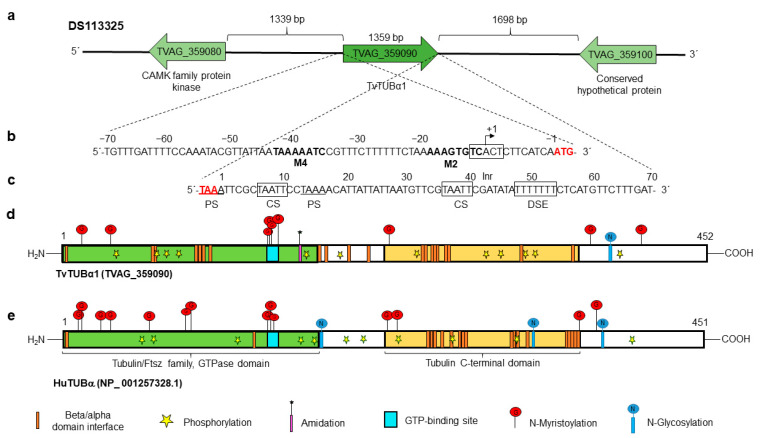
In silico analysis of the *tvtubα1* genomic context and regulatory regions and PTMs of the TvTUBα1 and HuTUB proteins. (**a**) Contig DS113325, TVAG_359090 corresponds to the *tvtubα1* gene (1359 nt) with intergenic regions of 1339 and 1698 bp containing the other genes (TVAG_359080 and TVAG_359100, respectively) present in the same contig. (**b**) 5′ 70 nt region upstream of the start codon (**ATG**) analyzed for regulatory elements. It presents initiator promoter elements (Inr), motif 2, and motif 4 (in bold letters). The +1 indicates the putative transcription start site. (**c**) 3′ 70 nt region downstream of the stop codon (**TAA**) analyzed for polyadenylation regulatory elements (black boxes). It has two putative polyadenylation signals (PSs), with the first coupled to the stop codon (red letters) and the other 14 nt downstream of the stop codon; one putative cleavage site (CS); and a T-rich downstream element (DSE). (**d**,**e**) Schemes of the TvTUBα1 and HuTUB proteins (TVAG_359090 and NP_001257328.1, respectively). These proteins have a tubulin C-terminal domain (cream box), a tubulin/Ftsz family GTPase domain (green box), a GTP-binding site (turquoise box), and multiple β/α domain interface sites (orange bars). Both proteins show putative PTMs, such as putative sites for phosphorylation (yellow star), N-glycosylation (blue circle with vertical line), and N-myristoylation (red circle with vertical line). However, only HuTUB presents putative amidation sites (purple bar with asterisk).

Moreover, the *tvtubα1* (TVAG_359090) gene is localized in the position 104561-106034 nt of the DS113325 contig flanked by a conserved hypothetical protein (TVAG_359080) and a CAMK family protein kinase (TVAG_359100). The 5′-intergenic region is 1339 nt long, and the 3′-intergenic region is 1698 nt long (Figure 2a). The in silico analysis of 70 nt up- and downstream of the *tvtubα1* ORF showed putative regulatory sequences for basal transcription and mRNA polyadenylation. The 5′-intergenic region contains a putative Inr (TCACT) located at −9 to −13 upstream from the initiation ATG codon of the *tvtubα1* gene. A motif 2 sequence (M2) with a consensus sequence at −12 to −19 nt (**AAAGTGTC**) and motif 4 sequence (M4) at −36 to −43 nt (**TAAAAATC**) was also found [49,50,51] (Figure 2b). The 3′-regulatory region of the *tvtubα1* gene has two putative polyadenylation signals (PSs) of **TAAA**. The first is included in the stop codon, and the other is located at +14 to +17 nt downstream of the stop codon. The gene also has one consensus TAATT cleavage site (CS) between nucleotides +7 and +11 and +37 and +41 after the stop codon and a T-rich (TTTTT) downstream element (DSE) between +49 and +55 nt (Figure 2c) [52,53]. The complete *tvtubα1* (TVAG_359090) gene (1359 nt) encodes a 452 aa protein (TvTUBα1) with a predicted molecular weight of ~50.1 kDa and a pI of 4.66. The human *hutub* (NP_001257328.1) gene taken as a reference encodes a 450 aa protein (HuTUB) with a molecular weight of ~50.4 kDa and a pI of 4.83. These proteins have 38.48% aa sequence identity. The in silico analysis showed that TvTUBα1 and HuTUB have one GTP-binding site (turquoise box), a tubulin/Ftsz family GTPase domain (green box), and a putative β/α domain interface (orange bars) (Figure 2d,e). TvTUBα1 and HuTUB have one and three putative sites for N-glycosylation, respectively. In TvTUBα1, the site is located at residue Asp380, and in HuTUB, the sites are located at Asp184, Asp337, and Asp370. In silico analysis also predicts putative N-myristoylation, in TvTUBα1 at residues Gly13, Gly34, Gly142, Gly143, Gly144, Gly146, Gly232, Gly365, and Gly412 and in HuTUB at positions Gly10, Gly13, Gly29, Gly34, Gly71, Gly83, Gly96, Gly98, Gly140, Gly141¸ Gly142, Gly144, Gly235, Gly244, Gly360, and Gly369. Regarding phosphorylation, TvTUBα1 presents 12 putative phosphorylation sites, Ser38, Ser94, Ser165, Ser193, Ser241, Ser287, Ser294, Ser398, Thr82, Thr73, Thr334, and Thr337. HuTUB presents 13 putative phosphorylation sites, Ser56, Ser75, Ser115, Ser172, Ser239, Thr178, Thr214, Thr221, Thr274, Thr285, Thr322, Thr409, and Thr429 (Figure 2d,e). Some differences were found, i.e., TvTUBα1 has a putative amidation site (Gly162), whereas HuTUB does not. However, these predicted PTMs should be taken with caution until they are experimentally demonstrated.

### 3.4. Expression of Bonafide Actin and Tubulin Genes and Actin-like and Tubulin-like Coding DNA Segments Reported in the Different ESTs and Proteomes of T. vaginalis

We reviewed and analyzed the different ESTs and proteomes reported thus far to search for evidence of bonafide actin and tubulin coding genes as well as actin-like and tubulin-like coding DNA segment expression [15,16,17,18,19,20,21,22,23,24,25,26,27,28,29,30]. At the transcriptional level, we observed that the 10 and 11 bonafide actin and tubulin coding gene products, respectively, showed the highest number of ESTs. (Supplementary Tables S1 and S2). The same effect was observed at the protein level during infection (Supplementary Tables S7 and S8).

Moreover, at the protein level, the bonafide actin and tubulin of group I with the highest identity were the most frequently identified in the different proteomes (Supplementary Tables S7 and S8). However, the concentration of actin expressing gene fragments was much higher in the trophozoite than in the cyst-like morphology. The opposite was true for tubulin-expressing gene fragments [15]. Interestingly, actin was more evident than tubulin during infection; however, these genes were differentially translated during infection [22], i.e., the *tvact1* gene was detected during the entire infection process, *tvact3* was not translated until 30 min after infection, and *tvact4* translation was turned on and off during infection, while *tvact10* was only detected at the beginning of infection. The differences observed in actin and tubulin expression showed that several tubulin genes were not expressed during infection. These data are consistent since actin is crucial in the ameboid morphology and plays an important role in *T. vaginalis* adherence and virulence, whereas tubulin is restructured in the cyst-like morphology. It was also found that the expression of these proteins was distinct, depending on the trichomonad isolate and culture conditions. In summary, the bonafide *tvact* and *tvtub* genes in group I were found in most of the proteomes analyzed, as evidence of expression at the protein level. Interestingly, some gene fragments encoding actin-like and tubulin-like domains in groups II-A and B showed some evidence of expression at the protein level. However, those belonging to group II-C were not found in any of the proteomes analyzed. Thus, these DNA sequences were considered as hypothetical proteins with actin-like or tubulin-like domains, respectively. Thus, these data show that the expression of the *tvact* and *tvtub* multigene families is complex (Supplementary Tables S1, S2, S7, and S8). 

### 3.5. Cloning and Sequencing of the tvact1 and tvtubα1 Genes and Expression and Purification of the Recombinant TvACT1 and TvTUBα1 Proteins for Antibody Production

**Figure 3:**
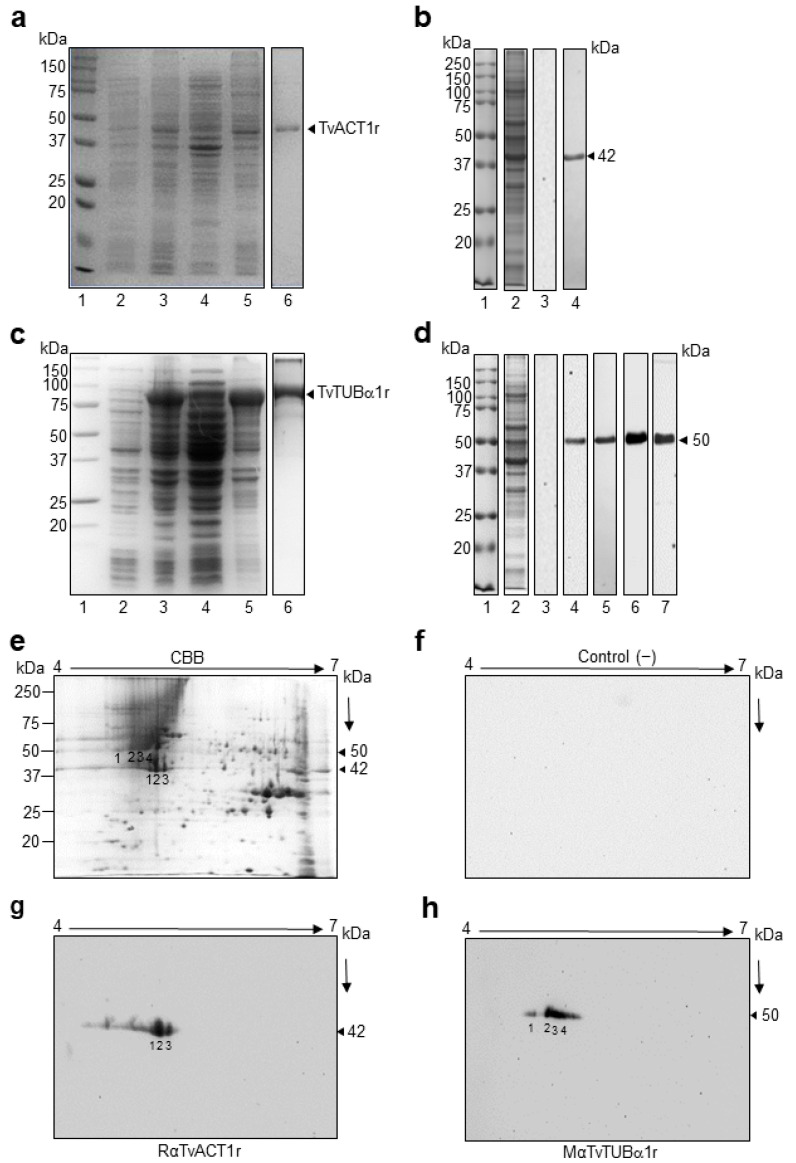
Purification and production of antibodies against the recombinant proteins TvACT1 and TvTUBα1 and recognition of TvACT and TvTUB trichomonad proteins. (**a**,**c**) Induction and purification of the TvACT1r and TvTUBα1r proteins, respectively. Lane 1, molecular weight marker; lane 2, uninduced bacterial extract; lane 3, induced extract; lane 4, soluble fraction; lane 5, insoluble fraction; and lane 6, purified TvACT1r and TvTUBα1r protein. (**b**,**d**) Western blot of anti-TvACT1r and TvTUBα1r homologous polyclonal antibody recognition and anti-TUB heterologous monoclonal antibodies in TCA extracts of *Tv.* Lane 1, molecular weight marker; lane 2, TCA extract; lane 3, secondary antibody used as a negative control; and lane 4 (panels (**b**,**d**)), recognition by the homologous anti-TvACT1r and heterologous anti-TUB antibody, respectively; panel d, lane 5, recognition by the homologous anti-TvTUBα1r antibody; lane 6, anti-TUBAcet antibody; and lane 7, anti-TUBPGlu antibody. (**e**) Trichomonad protein extract separated by 2DE in a 12% SDS-PAGE gel stained with Coomassie Brilliant Blue (CBB). (**f**) 2DE Western blot of a duplicate gel from panel e with the secondary antibody IgG-coupled to peroxidase (1:3000 dilution) used as a negative control. (**g**,**h**) 2DE WB of *Tv* extracts using rabbit anti-TvACT1r (1:3000 dilution) and mouse anti-TvTUBα1r (1:1000 dilution) antibodies. The line indicates the 4 to 7 pH range used for the IEF strips. The numbers in (**e**,**g***,***h**) indicate the spots identified by MS and recognized by rabbit anti-TvACT1r and mouse anti-TvTUBα1r antibodies.

To detect and localize bonafide TvACT and TvTUB in *Tv*, we first cloned, expressed, and purified recombinant TvACT1 and TvTUBα1 (TvACT1r and TvTUBα1r) proteins and produced polyclonal antibodies against them. By PCR assays, we amplified the complete 1131 bp *tvact1* and 1359 bp *tvtubα1* genes using genomic DNA from the CNCD 147 *Tv* clinical isolate as a template. The amplicons were cloned and sequenced (GenBank accession numbers: MZ695843 for actin and ON012693 for tubulin). The DNA sequences of the cloned *tvact and tvtub* genes showed 98% and 99% identity with the *tvact1* (TVAG_160060) and *tvtubα1* (TVAG_359090) genes found in the trichomonad genome sequence database (Supplementary Figures S3 and S4) [11]. The amplified *tvact1* gene showed 12 nt changes (A/G7, C/T93, C/T147, T/C156, C/T159, T/C177, T/C227, T/C660, A/C922, C/G924, C/A957, and C/T1119) (Supplementary Figure S3), which affected the aa sequence in two positions (K/E3 and N/Q308) (Supplementary Figure S5). In contrast, the amplified *tvtubα1* gene showed 13 nt changes (A/G45, C/T48, A/T78, A/G81, A/G93, C/T147, C/T186, T/G312, T/C813, C/T1089, C/T1122, and G/A1335). However, these changes did not affect the protein sequence (Supplementary Figure S6). These differences could be due to the distinct *Tv* isolates used. However, we could not discard that some of these changes resulted from errors during amplification, although we used high-fidelity polymerase. We used the CNCD147 Mexican *Tv* isolate, whereas the genome sequence was obtained from the G3 isolate [11]. The *tvact1* and *tvtubα1* amplicons were subcloned into the pCold1 and pCri1b vectors, respectively, and expressed in the *E. coli* BL21 (DE3) platform. After IPTG induction, a ~45 kDa band was obtained for TvACT1r with a polyhistidine tag, and a ~90 kDa band was obtained for TvTUB1αr, which has both a polyhistidine tag and an MBP tag with a molecular weight of ~40 kDa (Figure 3a,c, lane 5). The recombinant proteins were purified by affinity chromatography (Figure 3a,c, lane 6). Purified TvACT1r and TvTUBα1r were used as antigens for polyclonal antibody production in rabbits or mice (anti-TvACT1r and anti-TvTUBα1r, respectively) to detect and localize the bonafide actin and tubulin proteins in trichomonads by WB and indirect immunofluorescence assays.

### 3.6. Immunodetection of TvACT and TvTUB in T. vaginalis by 1DE and 2DE WB Assays

In the 1DE WB assay using trichomonad total protein extracts (Figure 3b,d, lane 2) obtained by TCA precipitation and transferred onto nitrocellulose (NC) membranes, the anti-TvACT1r antibody detected a single band with a molecular weight of ~42 kDa, which corresponded to the expected molecular weight of the bonafide TvACT1-TvACT10 proteins (Figure 3b, lane 4; Supplementary Table S1). The anti-TvTUBα1r antibody detected a single band with a molecular weight of ~50 kDa (Figure 3d, lane 5), similar to the heterologous monoclonal anti-TUB antibody (#13-8000, Invitrogen) (Figure 3d, lane 4). This molecular weight corresponded to that predicted in the in silico analysis for the group I bonafide tubulin (TvTUBα1-TvTUBα7 and TvTUBβ1-TvTUBβ4) proteins (Supplementary Table S2). Additionally, antibody recognition of tubulin with PTMs, such as acetylation (anti-TUBAcet) and polyglutamylation (anti-TUBPGlu), also showed a single ~50 kDa band (Figure 3d, lanes 6 and 7, respectively), which was similar to the results for the unmodified proteins. The secondary antibody used as a negative control showed no reaction, as expected (Figure 3b,d, lane 3). 

To identify the bonafide TvACT and TvTUB isoforms detected with the anti-TvACT1r and anti-TvTUBα1r antibodies in a TCA-total protein extract, we performed 2DE WB assays. The Coomassie blue-stained 2D gel pattern showed multiple protein spots in the molecular weight range between 15 and 250 kDa, with the majority near the neutral pI range (Figure 3e). In the 2DE WB assay, the anti-TvACT1r antibody recognized three ~42 kDa protein spots in the acidic pI range (Figure 3g, spots 1, 2, and 3). Anti-TvTUBα1r antibody recognized four ~50 kDa protein spots (Figure 3h, spots 1, 2, 3, and 4) that corresponded to the same abundant ~42 or ~50 kDa protein spots in the CBB-stained gel (Figure 3e, spots 1, 2, and 3 and spots 1, 2, 3, and 4, respectively), demonstrating a greater recognition of spot 2 with both antibodies. The secondary antibody used as a negative control showed no recognition, as expected (Figure 3f). The protein spots recognized by the specific anti-TvACT1r and anti-TvTUBα1r antibodies were identified by mass spectrometry (LC-ESI-MS). The results showed that the three spots recognized by the anti-TvACT1r antibody corresponded to the same gene product, TvACT4 (*tvact4*, TVAG_249200) (Table 1; Supplementary Figure S7). The three spots probably correspond to actin isoforms with different PTMs, such as phosphorylation, as predicted in the in silico analysis. The tubulin spots recognized by the anti-TvTUBα1r antibody were identified as TvTUBα1, TvTUBβ2, and TVAG_525430. TvTUBα1 was present in spots 1, 2, and 3, TvTUBβ2 was found only in spot 3, and TVAG_525430 was present in spots 2 and 4 (Table 1; Supplementary Figures S8–S11). Even though TvTUBα1 is very divergent from TvTUBβ2 and TVAG_525430, they share two conserved regions that are highly immunogenic, as predicted by in silico analysis; and structurally both proteins are very similar (Supplementary Figure S11). These could be the reason for the cross-recognition between these two tubulin isoforms by the anti-TvTUBα1r antibody. However, we could not discard that under the 2DE conditions used, these proteins migrated too close to be separated, as occurred with spots 1 and 4. Additionally, TvTUBβ2 and TVAG_525430 showed high sequence identity; however, this DNA segment encodes a truncated protein that lacks the first 29 and last 19 aa residues compared to TvTUBβ2 (Supplementary Figure S2). Interestingly all peptides identified in TVAG_525430 were found with identical aa sequence in TvTUBβ2. Therefore, we considered the identification of only two types of tubulins in the 50 kDa protein spots detected by the anti-TvTUBα1r antibody, one bonafide α and one bonafide β tubulin. This data may suggest the microtubule composition of the α and β isoforms in *Tv* under this culture conditions.

### 3.7. Immunolocalization of Actin and Tubulin in T. vaginalis

**Figure 4:**
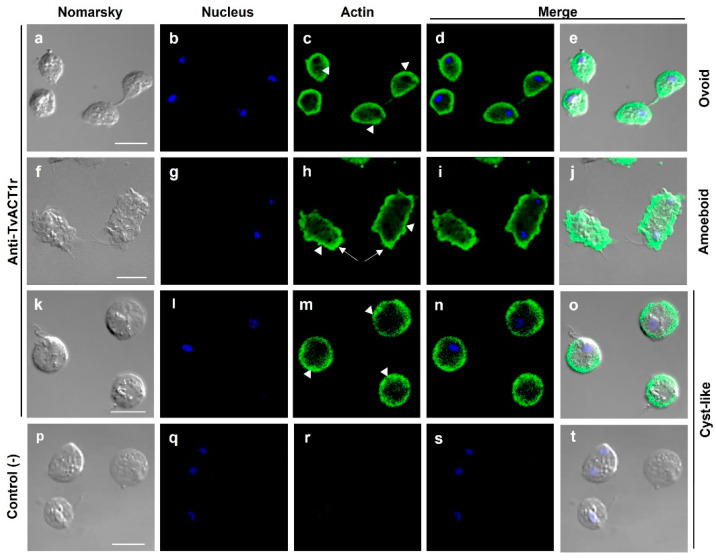
Localization of actin in different *T. vaginalis* morphologies. Ovoid (**a**–**e**), amoeboid (**f**–**j**), and cyst-like (**k**–**o**) parasites incubated with the anti-TvACT1r antibody and a FITC-coupled goat anti-rabbit secondary antibody; or only the secondary antibody used as a negative control (**p**–**t**). Nomarsky (**a**,**f**,**k**,**p**). Nuclei stained with DAPI ((**b**,**g**,**l**,**q**); in blue). Actin ((**c**,**h**,**m**); in green). Merged (**d**,**e**,**i**,**j**,**n**,**o**,**s**,**t**). Pseudopodia (arrows); mesh-like or accumulating actin (arrowheads). Bar: 10 µm.

To determine the localization of bonafide TvACT and TvTUB in *Tv*, IFAs were performed using the anti-TvACT1r and anti-TvTUBα1r antibodies on fixed and permeabilized parasites with different cellular morphologies. Figure 4 shows that the anti-TvACT1r antibody recognized actin in the cytoplasm, mainly in the periphery, in the three morphologies: ovoid (Figure 4a–e), amoeboid (Figure 4f–j), and cyst-like (Figure 4k–o), seen for cortical actin. Moreover, in amoeboid parasites, bonafide TvACT was concentrated in membrane projections or pseudopods, as expected (white arrows, Figure 4h). In the cyst-like structure, actin was concentrated in certain regions underneath the plasma membrane (arrowheads, Figure 4m). Actin was not observed in flagella or any other organelles, except in filopodia, between the two parasites (Figure 4c,e). Goat anti-rabbit secondary antibody used as a negative control showed no recognition, as expected (Figure 4p–t).

**Figure 5:**
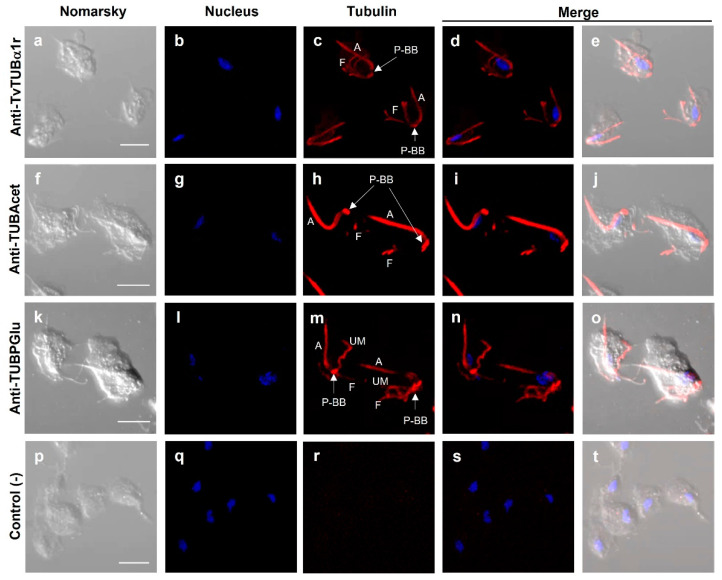
Localization of tubulin with different posttranslational modifications in *T. vaginalis*. Primary antibodies used: Anti-TvTUBα1r (**a**–**e**); anti-TUBAcet (anti-TUBAcetylated) (**f**–**j**); anti-TUBPGlu (anti-TUBPolyglutamylated) (**k**–**o**); only the secondary antibody used as a negative control (**p**–**t**). DAPI for nucleus staining ((**b**,**g**,**l**,**q**); in blue) and Nomarsky images (**a**,**f**,**k**,**p**). A: axostyle; F: flagella; P–BB: pelta–basal bodies complex; UM: undulating membrane; P–BB–PA: pelta–basal bodies–posterior axostyle complex. Scale bar: 10 µm. No paradesmose structure was detected with these antibodies in parasites in interphase.

**Figure 6:**
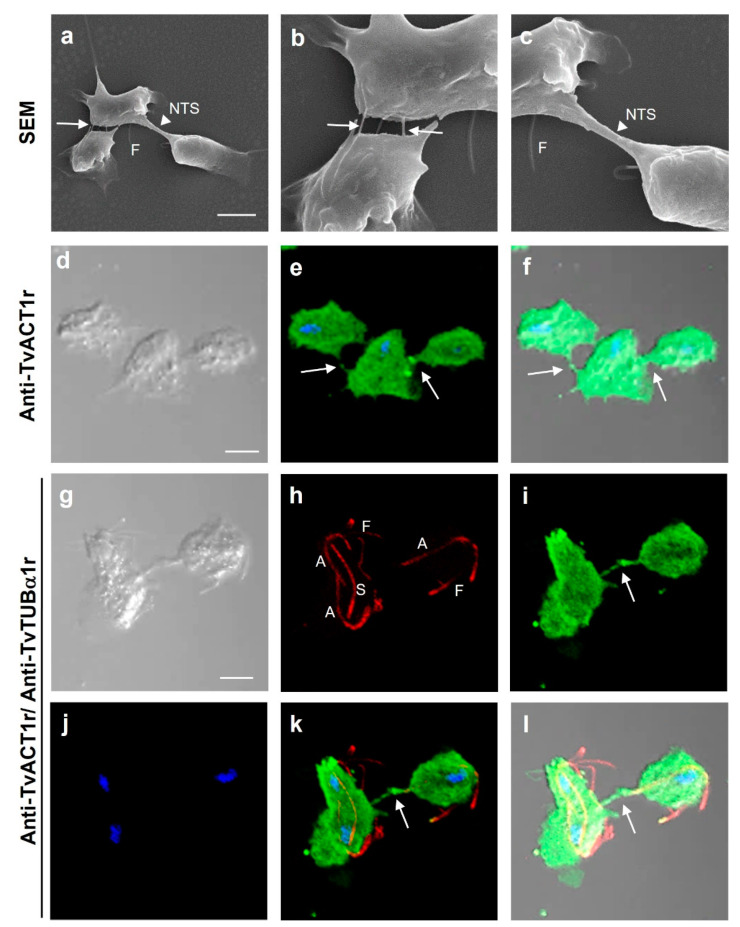
Different interactions in *T. vaginalis.* (**a**–**c**) SEM of the parasite–parasite interactions through filopodia-like structures (white arrow) and during cell division through nanotube-like structures (NTS; white arrowhead). (**d**–**f**) IFA to detect the presence of actin in cell–cell interactions using anti-TvACT1r antibody. (**g**–**l**) IFA of cell–cell interactions using anti-TvACT1r and anti-TvTUBα1r antibodies, including the mitotic spindle (S). Panels **b** and **c** are a magnified view of panel **a**. Nomarsky (**d**,**f**,**j**,**l**); merged (**e**,**f**,**k**,**l**); DAPI-stained nuclei (blue); tubulin with the polyclonal anti-TvTUBα1r antibody (**h**,**k**,**l**); actin with the polyclonal anti-TvACT1r antibody (**e**,**f**,**i**,**k**,**l**). NTS: nanotube-like structure; A: axostile; F: flagella; S: spindle; arrows: presence of actin in cell–cell interactions. Scale bar: 10 µm.

Figure 5 and Figure 6 show that the anti-TvTUBα1r antibody recognized tubulin in the axostyle (A), pelta–basal body (P–BB) complex, flagella (F) (Figure 5c,d,e), and extra microtubular structure dubbed paradesmose (S) (Figure 6h,k,l). This structure is considered an extranuclear mitotic spindle in *Tv,* a structure that has not been reported in *Tritrichomonas foetus* [39,55]. Moreover, the recognition of tubulin PTMs by antibodies was also demonstrated. The anti-TUBAcet antibody showed strong recognition in A, the P–BB complex, and F (Figure 5h,i,j). In contrast, the anti-TvTUBPGlu antibody recognized A, the P–BB complex, and the undulating membrane (UM) but not F (Figure 5m,n,o). These recognition patterns agreed with previously reported data [32,33,34,35,36,37,38,39,40]. Goat anti-mouse secondary antibody used as a negative control showed no recognition, as expected (Figure 5p–t).

### 3.8. Different Types of Cell–Cell Interactions Mediated by Actin or Tubulin in T. vaginalis

Just as *Tv* interacts with the host cell, it is also capable of parasite–parasite interactions. To visualize different types of cell–cell interactions among parasites and to show the types of cytoskeleton proteins involved, scanning electron microscopy (SEM) images were obtained and analyzed. To differentiate actin-containing structures from those containing tubulin, IFAs were also performed (Figure 6). By SEM, it was possible to observe cell–cell interactions through filopodia-like (white arrows) and nanotube-like structures (NTS; white arrowhead) (Figure 6a–c). By IFA, both actin- and tubulin-containing structures were observed and differentiated during interphase and mitosis (Figure 6d–l). Parasites in interphase and a dividing parasite interacting with another parasite in interphase showed the presence of bonafide actin in filopodia-like cell–cell interactions (Figure 6d–l). In a dividing parasite, tubulin-containing structures were also observed in the cytoplasm, including a spindle-like structure dubbed paradesmose (S), in addition to the axostyles (A) and flagella (F) (Figure 6h,k,l). 

### 3.9. Immunolocalization of Both TvACT and TvTUB in T. vaginalis during Karyokinesis and Cytokinesis

**Figure 7:**
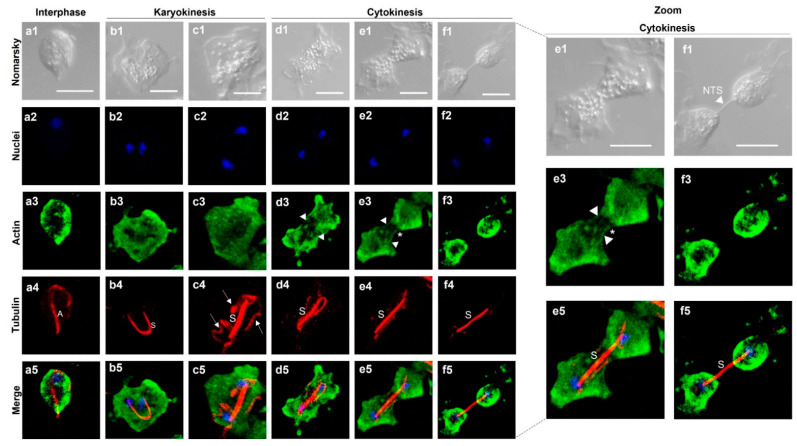
Localization of bonafide TvACT and TvTUB during *T. vaginalis* karyokinesis and cytokinesis. Indirect immunofluorescence assay using anti-TvACT1r ((**a3**–**f3**); in green) and anti-TUB ((**a4**–**f4**); in red) homologous and heterologous antibodies, respectively, and DAPI for nuclear staining ((**a2**–**f2**); in blue). Nomarsky images (**a1**–**f1**). (**a1**–**a5**) Parasite in interphase; (**b1**–**b5**,**c1**–**c5**) parasite in karyokinesis; (**d1**–**d5**, **e1**–**e5**,**f1**–**f5**) parasite in cytokinesis; (**a5**–**f5**) signal merging of TvACT and TvTUB during mitosis. Dotted lines indicate magnified views of parasites in cytokinesis. A: axostyle; S: mitotic spindle dubbed paradesmose; white arrows: other tubulin-containing structures; white arrowheads: actin fibers; (*): membrane strangulation during cytokinesis. NTS: nanotube-like structure. Scale bar: 10 µm.

To observe the localization of actin and tubulin during the late stages of *Tv* mitosis, we induced mitosis by incubating the parasites in cold medium and then the spent medium was replaced with fresh medium to perform IFAs with anti-TvACT1r and heterologous monoclonal anti-TUB antibodies. Figure 7 shows that during interphase, in an ovoid parasite, a single nucleus (in blue), tubulin in A and the P–BB complex (in red), and actin (in green) at the periphery of the parasite were detected (Figure 7(a1–a5)). In karyokinesis, the presence of two nuclei and the appearance of the mitotic spindle (U-shaped paradesmose; S) were observed (Figure 7(b1–b5)). Paradesmose appeared attached to nuclei ends, and actin was concentrated in the cytoplasm and parasite pseudopods. Paradesmose was generally seen as a U-shaped structure that was later longitudinally aligned, maintaining one nucleus at each end. Other tubulin-containing structures were also seen (arrows) (Figure 7(c1–c5)). 

In cytokinesis, the parasite became amoeboid and elongated, the aligned tubulin-containing paradesmose lengthened, and the formation of actin stress fibers during membrane strangulation of the dividing cells to separate the daughter cells was also observed (Figure 7(d1–d5,e1–e5)). However, the typical contractile ring was not observed. Actin was also concentrated in the cytoplasm. At this stage, it was possible to observe structures in the form of stress fibers (arrowheads) concentrated at the parasite ends, displaying the extended actin cytoskeleton (Figure 7(d1–d5,e1–e5)). Cytokinesis is the last process of mitosis. The separation of individual ovoid daughter cells containing their own actin cytoskeletons and plasma membrane (Figure 7(f1–f5)) was also observed. However, it is surprising that the tubulin-containing paradesmose structure kept the daughter cells together through a nanotube-like membranal structure, despite the separation of the cytoplasm and membrane, as can be better appreciated in the magnified cytokinesis stage images. 

Based on these results, different cell–cell interactions can be observed in *T. vaginalis* in interphase and during mitosis, such as filopodia-like structures in which actin is present and nanotube-like structures that contain tubulin, which was only observed in dividing parasites during the last stages of mitosis (Figure 6 and Figure 7).

### 3.10. Last Stage of T. vaginalis Cytokinesis Observed by SEM Analysis

**Figure 8:**
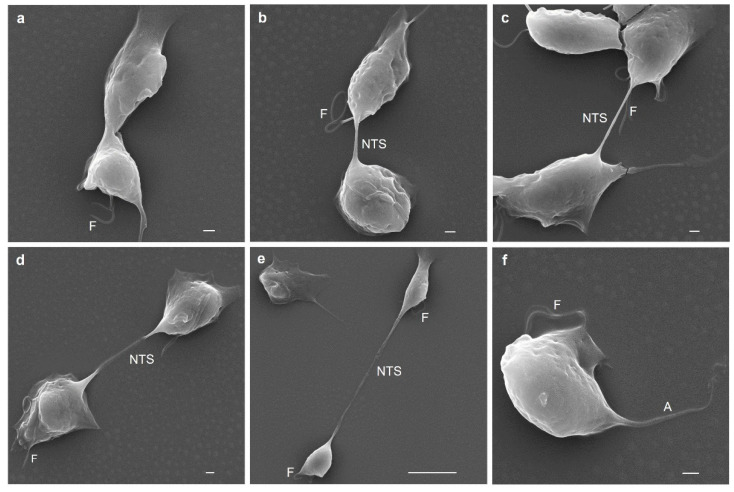
Scanning electron microscopy (SEM) of cytokinesis in *T. vaginalis*. Initiation cytokinesis in *Tv* (**a**); presence of a nanotube-like structure (NTS) where paradesmose was found by IFA (Figure 7) (**b**); lengthening of the NTS (**c**); the NTS becoming electron lucent during lengthening (**d**); the NTS structure thinned and broke (**e**); newly divided parasite (**f**). A: axostile; NTS: nanotube-like structure; F: flagella.

To observe the last stage of cytokinesis in more detail, SEM analysis was performed to follow the nanotube-like paradesmose-containing structure in the last stage of cytokinesis (Figure 8). In this last stage, the axostyle was no longer visualized, and only the flagella and the strangulation of the dividing cell were observed (Figure 8a), as also visualized by IFA (Figure 7). As the daughter cells stretched with the help of the actin cytoskeleton (Figure 7), the NTS was elongated and thinned (Figure 8b,c). This nanotube-like structure became translucent beginning at its middle section and adhered to the surface when parasites were at the maximum point of the division (Figure 8d) before the structure thinned and broke (Figure 8e). This NTS has a variable length, depending on the elongation process, that ranges from ~7 to 32 µm and a diameter of 300–500 nm, which are similar to those reported for different types of intercellular TNTs [42,43,44,45]. The tension exerted in the elongation process to achieve the final separation of the daughter cells caused the NTS to break and promoted the daughter cells to swim free from one another (Figure 8f). The axostyle and flagellum of *Tv* were observed again in the divided parasite in interphase (Figure 8f). Thus, each daughter cell was able to initiate a new cell cycle.

## 4. Discussion

Actin and tubulin genes vary among eukaryotic organisms; however, at the structural level, they are highly conserved [56]. Each actin and tubulin isoform has a specific function as well as a subcellular location [57,58] and plays important roles in the dynamics and organization of the cytoplasm during cell division and cell motility. The actin and tubulin genes in *Tv* are part of the multigene families of this parasite, which has 10 bonafide actin and 11 bonafide tubulin genes [10,11], as occurs with actin in *Giardia* (https://giardiadb.org/giardiadb/app, accessed on 9 May 2022), *Dictyostelium discoideum,* and *Arabidopsis thaliana* [59,60], as well as with the tubulin superfamily in higher organisms such as *Drosophila* [61,62]. Interestingly, *Tv* also have 19 and 20 DNA segments coding some domains with homology to typical actin or tubulin proteins, in addition to other functional domains that do not fulfill the characteristics of complete expressing genes. These sequences form two groups. Group I encodes proteins with high identity to the typical actin or tubulin proteins, with 10 actin genes and 11 tubulin genes (7 α and 4 β type), in these groups, suggesting gene duplication events [59]. Group II is formed by all the other DNA segments that encode actin-like or tubulin-like domains, which differ in their amino acid sequences or are truncated products. These were considered actin-like, tubulin-like, or hypothetical proteins, respectively. These data probably indicate that the genome contigs are not yet properly assembled or are products generated by repeated sequences of the genome of *Tv*. 

The actin and tubulin *Tv* transcriptomic and proteomic data showed their differential expression among isolates and environmental conditions. These data suggest the complexity of the regulation of the expression of actin and tubulin isoforms. However, the question is why evolution would allow this feature that would imply a high energy expenditure Perhaps the dynamics of the actin and tubulin cytoskeleton in *Tv* may be due to different PTMs or their interaction with microtubule-associated proteins, as occurs in other actin and tubulin families [63,64,65]. 

In this study, we demonstrated that the anti-TvACT1r and anti-TvTUBα1r homologous polyclonal antibodies recognized in 1DE WB assays, which included a single protein band of the expected size for bonafide TvACT and TvTUB corresponded to three and four spots, respectively, in 2DE WB assays. Interestingly, the identification of actin spots numbered 1–3 (Figure 3g) by mass spectrometry indicated TvACT4 (TVAG_249200) instead of TvACT1 (TVAG_160060). This could be due to the cross-reactivity of the anti-TvACT1r antibody with any of the ten TvACT proteins of the high-identity group (99 to 100%) (Supplementary Table S1), suggesting that TvACT4 is the protein most highly expressed in this isolate and under our working conditions that may undergo posttranslational modifications such as phosphorylation. As occurs in *Dictyostelium,* the phosphorylation of Tyr-53 modifies its pI and could be an important actin regulatory mechanism [66,67,68]. 

Previous proteomic studies have reported the presence of TvACT1, TvACT2, TvACT5, TvACT8, and TVAG_371880 in different protein spots [16,17,20]. Thus, TvACT4 joins this list of actin proteins identified in distinct *Tv* proteomes. These results agreed with those reported in TrichDB, since these 10 bonafide *tvact* gene products presented the highest EST numbers and belonged to group I with the greatest identity in the aa sequence, except TVAG_371880, which belongs to group II-A. This indicates that TvACT proteins are differentially expressed according to the isolate, culture conditions, and virulence. The expression of multiple actin genes in *Tv* may be part of the adaptability of the pathogenesis mechanisms of this parasite that allows quick responses to different stimuli, i.e., amoeboid morphogenesis during infection [6,10,22], stress temperature, the presence or absence of nutrients: iron, zinc, putrescine, glucose, etc. 

Regarding tubulin, its expression is also divergent. However, the expression between the high- and low-identity groups was somewhat similar (Supplementary Table S4). This is supported by our results, since the anti-TvTUBα1r antibody recognized two proteins of the bonafide group I TvTUBα1 and TvTUBβ2 with similar molecular weights of ~50 kDa. In contrast with those obtained by Huang et al. [20], who reported the differential expression of TvTUBα2 (group I) and TVAG_148390 (group II-B) from the divergent group of tubulin-like proteins between the ovoid and amoeboid forms, which presented different molecular weights and pI values. The differences in size and pI may be due to changes in PTMs, such as acetylation or polyglutamylation [35,36,37,39]. Together, our data and those from the different proteomes indicate the differential expression of distinct TvACT-like and TvTUB-like protein fragments among *Tv* isolates, working conditions, and complex formation [18,19,20,21,22,23,24,26,27,28,29,30]. 

The distribution of TvACT in the ovoid, amoeboid, and cyst-like morphologies of *Tv* determined using the anti-TvACT1r antibody was similar, with TvACT concentrated on the periphery of the parasite but mainly on the pseudopods, or cytoplasmic extensions, filopodia, and in the ectoplasmic fringe in the amoeboid form. This is consistent with reports by Brugerolle’s, Liu’s, and Cappuccinelli’s groups [31,66,69]. A mesh-like structure, which may have contained actin filaments, was also observed mainly in the amoeboid morphology, as has been reported in other organisms [66,70]. In dividing parasites, the anti-TvACT1r antibody recognized some actin stress fibers during cytokinesis, suggesting that actin is present during this process. However, the typical contractile ring was not detected. Moreover, the heterologous anti-tubulin antibody also used in this study recognized both the axostyle and paradesmose, which is a microtubular structure that actively participates in mitosis in *Tv* [32,39,71]. IFA and SEM data indicate that actin and tubulin are present in parasite–parasite interactions, suggesting intercellular communications in *Tv* tunneling nanotube-like structures (TNTs)*,* as it happens in other cells [42,43,44,45]. Surprisingly, paradesmose held the daughter cells together until the complete separation of the parasites occurred, despite already appearing separated in terms of the cytoplasm and plasma membrane. These data suggest that this NTS paradesmose-containing structure observed by SEM and confocal microscopy is important during cytokinesis in *Tv* and could contain and carry functions similar to the tunneling nanotubes. These structures have variable diameters, depending on their composition. Those of actin have a diameter of ~50 to <500 nm and those that contain tubulin have a range of ~400 to 1500 nm [72]. The TNT-like structures observed in *Tv* have a diameter of ~300–500 nm, which agrees with that tubulin was found in this NTS (Figure 7(f1–f5)). This has been reported in several cell types for transporting proteins, RNA, or even organelles, and its diameter will depend on what they transport. This structure in *Tv* probably may help to balance the cellular content between the daughter cells before complete separation. TNTs containing tubulin have been reported to be diverse in morphology and functions, and participate in intercellular communication in primary neurons, astrocytes, and human monocyte-derived macrophages [72], as shown in this study for *Tv*. 

## 5. Conclusions

In conclusion, the expression of different TvACT and TvTUB proteins is very complex and both protein types are present in specific intercellular interactions in trichomonads. Thus, future work should be directed to detect the presence of distinct PTMs and their effects on the morphogenesis of *Tv* and associations with actin-binding proteins in each of the TvACTs and TvTUBs under different environmental conditions. It will also be interesting for future work to characterize the role of nanotube-like paradesmose-containing structures during cytokinesis in *Tv* and to identify the type of molecules and possible organelles that could travel through this type of tunnels of intercellular communication in the last stage of trichomonal cellular division. 

## Figures and Tables

**Table 1:** Proteins identified by LC/ESI/MS of spots detected by anti-TvACT1r and anti-TvTUBα1r antibodies in 2DE WB assays.

Antibody	Spot Code ^1^	Protein Type ^2^	Locus Tag ^3^ TVAG_	Protein Number ^4^	Coverage (%) ^5^	Theoretical Molecular Mass (kDa)
anti-TvACT1r	1	Actin	249200	TvACT4	54.52	42.2
	2	Actin	249200	TvACT4	54.52	42.2
	3	Actin	249200	TvACT4	42.55	42.2
anti-TvTUBα1r	1	α-tubulin	456920	TvTUBβ2	15.21	50.5
	2	β-tubulin	359090	TvTUBα1	24.56	50.7
		γ-tubulin	525430/456920	TvTUBβ2	25.88/23.00	45.2/50.5
	3	α-tubulin	456920	TvTUBβ2	34.68	50.5
		β-tubulin	359090	TvTUBα1	20.13	50.7
	4	γ-tubulin	525430/456920	TvTUBβ2	25.88/23.00	45.2/50.5

^1^ Spot code corresponds to protein spot numbers shown in 2DE WB (Figure 3). ^2^ Protein name in the genome of *Tv* (https://trichdb.org/trichdb/app, accessed on 29 May 2022). ^3^ Identification tag of the gene in the *Tv* genome sequence. ^4^ Protein number assigned to bonafide actin and tubulin proteins shown in Supplementary Tables S1 and S2. ^5^ Protein coverage of the peptides obtained by LC/ESI/MS. The in silico analysis and the experimental data allow us to reclassify the identified tubulins as α and β tubulins.

## Data Availability

Data are contained within the article or Supplementary Materials. Additional data presented in this study are available on request to the corresponding author.

## Supplementary Materials

The following are available online at https://www.mdpi.com/article/10.3390/genes13061067/s1, Table S1. Analysis of DNA sequences encoding actin-like protein segments found in the *T. vaginalis* genome database, Table S2: Analysis of DNA sequences encoding tubulin-like protein segments found in the *T. vaginalis* genome database, Table S3: Multiple alignment of the 5′ and 3′ regulatory sequences of the bonafide actin-encoding genes of *T. vaginalis,* Table S4: Multiple alignment of the 5′ and 3′ regulatory sequences of the bonafide tubulin-encoding genes of *T. vaginalis,* Table S5: Identification of the putative regulatory elements and position at the 5′ and 3′ regulatory regions of the DNA sequences encoding actin-like protein fragments found in the *T. vaginalis* genome database, Table S6: Identification of the putative regulatory elements and position at the 5′ and 3′ regulatory regions of the DNA sequences encoding tubulin-like protein fragments found in the *T. vaginalis* genome database, Table S7: Protein expression of actin-like encoding gene fragments in proteomes reported of different *T. vaginalis* isolates and under distinct culture conditions, Table S8: Protein expression of tubulin-like gene fragments in proteomes reported of different *T. vaginalis* isolates and under distinct culture conditions, Table S9: Predicted 3D structures of actin-like aa deduced sequences found in the *T. vaginalis* genome database, Table S10: Predicted 3D structures of tubulin-like aa deduced sequences found in the *T. vaginalis* genome database. Figure S1: In silico analysis of the 29 actin-like deduced aa sequences reported in the *T. vaginalis* genome database, Figure S2: In silico analysis of the 31 tubulin-like deduced aa sequences reported in the *T. vaginalis* genome database, Figure S3: Nucleotide sequence alignment between the *tvact1* gene amplified from the *T. vaginalis* CNCD147 isolate and the one from clone G3 from the *T. vaginalis* genome, Figure S4: Nucleotide sequence alignment between the *tvtub1 tvtub**α1* gene amplified from the *T. vaginalis* CNCD147 isolate and the one from the G3 clone from the *T. vaginalis* genome, Figure S5: Amino acid alignment of the TvACT1 protein of the *T. vaginalis* CNCD147 isolate and the G3 *T. vaginalis* genome, Figure S6: Amino acid alignment of the TvTUBα1 protein of the *T. vaginalis* CNCD147 isolate and the one from the G3 clone of the *T. vaginalis* genome, Figure S7: Localization of peptides identified by LC-ESI-MS of the spot, 1–3 of actin protein (TvACT4; TVAG_249200), Figure S8: Localization of peptides identified by LC-ESI-MS of the spot 1 and 3 of tubulin protein (TvTUBβ2; TVAG_456920), Figure S9: Localization of peptides identified by LC-ESI-MS of the spot 2 and 3 of tubulin protein (TvTUBα1; TVAG_359090), Figure S10: Localization of peptides identified by LC-ESI-MS of the spot 2 and 4 of tubulin protein (TVAG_525430), Figure S11: Alignment and modeling of TvTUBα1 and TvTUBβ2 identified by LC-ESI-MS.
