# Supplementary material for: Omics Analyses of Trichomonas vaginalis Actin and Tubulin and Their Participation in Intercellular Interactions and Cytokinesis
Source: Genes (Basel). 2022 Jun 15;13(6):1067. doi: 10.3390/genes13061067 (PMC9222396; doi:10.3390/genes13061067)
Supplement: Supplementary file 1 [file Supplementary Figure S4.pdf › sentGalley Suppl. FIGURES-100622/Supplementary Figure S4.pdf]

```
CNCD147   ATGCGTGAAGTTATTTCTATCCACGTTGGACAGGCCGGTGTTCAAATCGGTAACGGCTGCTGGGAACTCTACTGCCTAGA 80
G3        ATGCGTGAAGTTATTTCTATCCACGTTGGACAGGCCGGTGTTCAGATTGGTAACGGCTGCTGGGAACTCTACTGCCTTGA 80
          ********************************************.** *****************************:**

CNCD147   ACACGGTATCCAACCAGATGGCCAACTCCCATCAGACAAGACAATCGGTGTCGAAGATGATGCTTTCAACACATTCTTCT 160
G3        GCACGGTATCCAGCCAGATGGCCAGCTCCCATCAGACAAGACAATCGGTGTCGAAGATGATGCTTTTAACACATTCTTCT 160
          .***********.***********.***************************************** *************

CNCD147   CCGAGACAGGCGCCGGCAAGCACGTCCCACGTGCTGTTTTCGTCGATCTTGAACCAACAGTCGTCGACGAAGTCCGCACA 240
G3        CCGAGACAGGCGCCGGCAAGCACGTTCCACGTGCTGTTTTCGTCGATCTTGAACCAACAGTCGTCGACGAAGTCCGCACA 240
          ************************* ******************************************************

CNCD147   GGCACATACCGCCAGCTTTTCCACCCAGAGCAGCTCATCTCCGGCAAGGAAGATGCCGCTAACAACTACGCTCGTGGCCA 320
G3        GGCACATACCGCCAGCTTTTCCACCCAGAGCAGCTCATCTCCGGCAAGGAAGATGCCGCTAACAACTACGCGCGTGGCCA 320
          *********************************************************************** ********

CNCD147   CTACACAGTTGGTAAGGAAATCATCGATCTCACACTCGACCGCATCCGTAAGCTTGCTGATCAGTGCACAGGTCTTCAGG 400
G3        CTACACAGTTGGTAAGGAAATCATCGATCTCACACTCGACCGCATCCGTAAGCTTGCTGATCAGTGCACAGGTCTTCAGG 400
          ********************************************************************************

CNCD147   GCTTCCTTATCTTCCACTCCTTCGGTGGTGGCACAGGCGCTGGCTTCGGCTCCCTCCTTCTCGAGCGTCTTTCTGTCGAC 480
G3        GCTTCCTTATCTTCCACTCCTTCGGTGGTGGCACAGGCGCTGGCTTCGGCTCCCTCCTTCTCGAGCGTCTTTCTGTCGAC 480
          ********************************************************************************

CNCD147   TACGGCAAGAAGTCCAAGCTCGAATTCACAGTCTACCCATCTCCACAGGTTTCCACAGCCATCGTCGAGCCATACAACTC 560
G3        TACGGCAAGAAGTCCAAGCTCGAATTCACAGTCTACCCATCTCCACAGGTTTCCACAGCCATCGTCGAGCCATACAACTC 560
          ********************************************************************************

CNCD147   CATTCTCGCTACACACTCCATGATCGATCACTCCGACTGCGCTTTCATGGTCGATAACGAAGCTCTCTACGATCTCTGCC 640
G3        CATTCTCGCTACACACTCCATGATCGATCACTCCGACTGCGCTTTCATGGTCGATAACGAAGCTCTCTACGATCTCTGCC 640
          ********************************************************************************

CNCD147   GCCGTGCCCTCGATATCGAGCGCCCAACATACACCAACCTCAACCGTCTCATCGGCCAGGTTGTTTCCTCCCTCACAGCC 720
G3        GCCGTGCCCTCGATATCGAGCGCCCAACATACACCAACCTCAACCGTCTCATCGGCCAGGTTGTTTCCTCCCTCACAGCC 720
          ********************************************************************************

CNCD147   TCCCTCAGATTCGATGGTGCCCTCAACGTCGACTTCACAGAGTTCCAGACAAACCTTGTCCCATACGCTCGTATCCACTT 800
G3        TCCCTCAGATTCGATGGTGCCCTCAACGTCGACTTCACAGAGTTCCAGACAAACCTTGTCCCATACGCTCGTATCCACTT 800
          ********************************************************************************

CNCD147   CCCAATCTGCTCTTATGCTCCAGTCATTTCTGCAGAGAAGGCCTACCACGAGCAGCTCTCTGTTGCCGAAATCACAAACT 880
G3        CCCAATCTGCTCCTATGCTCCAGTCATTTCTGCAGAGAAGGCCTACCACGAGCAGCTCTCTGTTGCCGAAATCACAAACT 880
          ************ *******************************************************************

CNCD147   CACTCTTCGAGCCAGCCAACATGATGGTTAAGTGCGACCCACGCCACGGCAAGTACATGGCTTGCACACTCCTTTACCGT 960
G3        CACTCTTCGAGCCAGCCAACATGATGGTTAAGTGCGACCCACGCCACGGCAAGTACATGGCTTGCACACTCCTTTACCGT 960
          ********************************************************************************

CNCD147   GGTGATGTCGTTCCAAAGGATGTCTCCGCTGCTGTCGCTACAATCAAGACAAAGCGCACAATCCAGTTCGTCGACTGGTG 1040
G3        GGTGATGTCGTTCCAAAGGATGTCTCCGCTGCTGTCGCTACAATCAAGACAAAGCGCACAATCCAGTTCGTCGACTGGTG 1040
          ********************************************************************************

CNCD147   CCCAACAGGCTTCAAGATGGGTATCAACTACCAGCCACCAACAGTCGTCCCAGGTGGCGACCTCGCAAAGGTCCAGCGCG 1120
G3        CCCAACAGGCTTCAAGATGGGTATCAACTACCAGCCACCAACAGTCGTTCCAGGTGGCGACCTCGCAAAGGTCCAGCGCG 1120
          ************************************************ *******************************

CNCD147   CCGTCTGCATGCTCGCTAACACAACAGCCATCGCCGAAGCTTGGTCTCGCCTCGACCACAAGTTCGACCTCATGTACGCT 1200
G3        CTGTCTGCATGCTCGCTAACACAACAGCCATCGCCGAAGCTTGGTCTCGCCTCGACCACAAGTTCGACCTCATGTACGCT 1200
          * ******************************************************************************

CNCD147   AAGCGTGCTTTCGTCCACTGGTACGTCGGTGAGGGTATGGAAGAAGCTGAATTCCCAGAAGCTCGTGAAGATCTTGCCCT 1280
G3        AAGCGTGCTTTCGTCCACTGGTACGTCGGTGAGGGTATGGAAGAAGCTGAATTCCCAGAAGCTCGTGAAGATCTTGCCCT 1280
          ********************************************************************************

CNCD147   CCTCGAGAAGGACTACGACGAAGTCGCTGCTGAATCAGTCGAAGGTGGCGATGAGGAAGAAGATGGTGGCGAAATGTAA 1359
G3        CCTCGAGAAGGACTACGACGAAGTCGCTGCTGAATCAGTCGAAGGTGGCGATGAAGAAGAAGATGGTGGCGAAATGTAA 1359
          ******************************************************.************************
```

Supplementary Figure S4. Nucleotide sequence alignment between the *tvtubα1* gene amplified from the *T. vaginalis* CNCD147 isolate and G3 from the *T. vaginalis* genome. The letters in red indicate the nt changes found after the amplicon DNA sequencing. * Represents identical nucleotides.
