# Supplementary material for: Omics Analyses of Trichomonas vaginalis Actin and Tubulin and Their Participation in Intercellular Interactions and Cytokinesis
Source: Genes (Basel). 2022 Jun 15;13(6):1067. doi: 10.3390/genes13061067 (PMC9222396; doi:10.3390/genes13061067)
Supplement: Supplementary file 1 [file genes-13-01067-s001.zip › sentGalley Suppl. FIGURES-100622/Supplementary Figure S1.pdf]

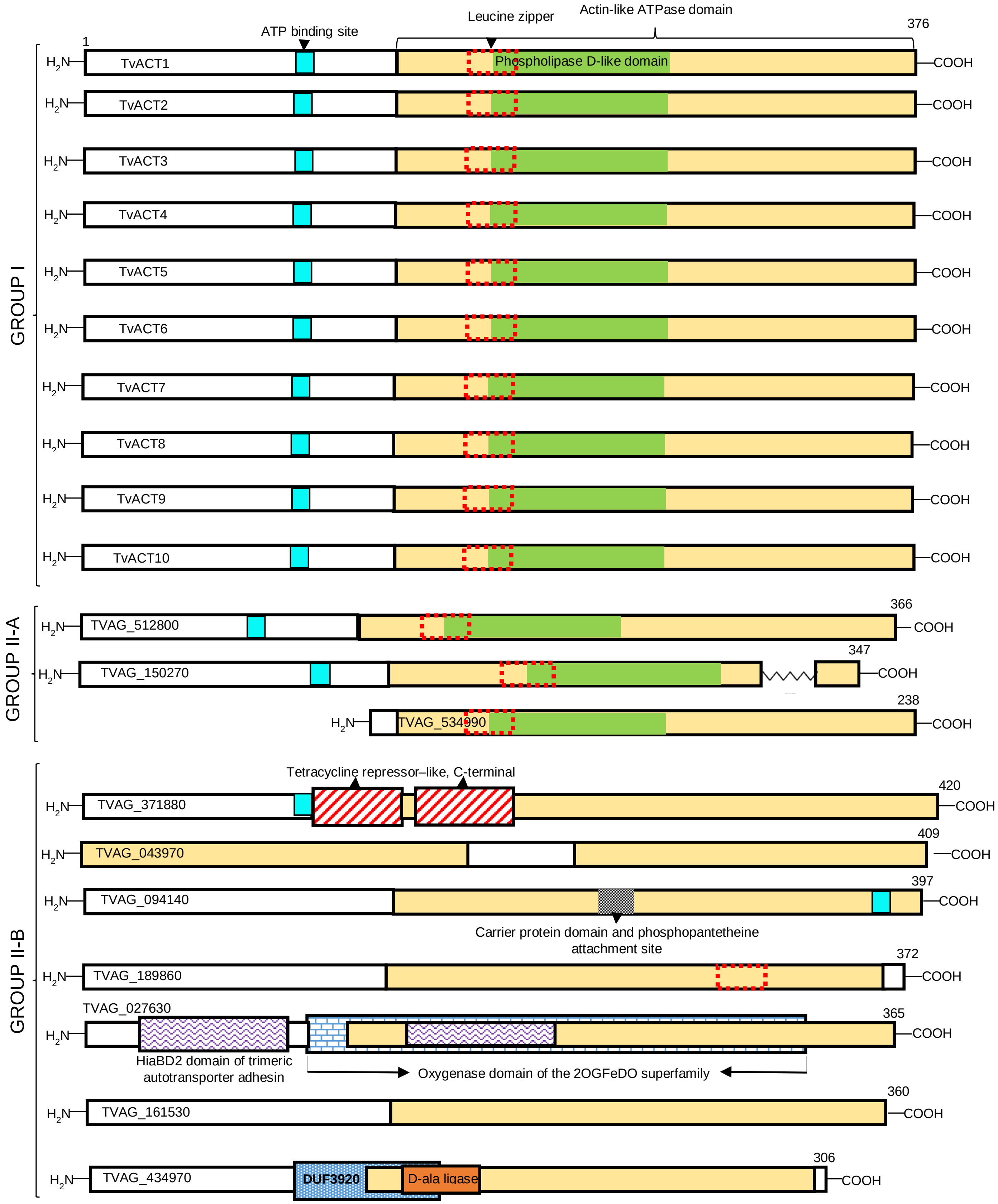

GROUP I
ATP binding site
Leucine zipper
Actin-like ATPase domain
1
376
Phospholipase D-like domain
H_2N TvACT1 COOH
H_2N TvACT2 COOH
H_2N TvACT3 COOH
H_2N TvACT4 COOH
H_2N TvACT5 COOH
H_2N TvACT6 COOH
H_2N TvACT7 COOH
H_2N TvACT8 COOH
H_2N TvACT9 COOH
H_2N TvACT10 COOH
GROUP II-A
366
H_2N TVAG_512800 COOH
347
H_2N TVAG_150270 COOH
238
H_2N TVAG_534090 COOH
GROUP II-B
Tetracycline repressor–like, C-terminal
420
H_2N TVAG_371880 COOH
409
H_2N TVAG_043970 COOH
397
H_2N TVAG_094140 COOH
Carrier protein domain and phosphopantetheine attachment site
372
H_2N TVAG_189860 COOH
TVAG_027630
365
H_2N COOH
HiaBD2 domain of trimeric autotransporter adhesin
Oxygenase domain of the 2OGFeDO superfamily
360
H_2N TVAG_161530 COOH
306
H_2N TVAG_434970 COOH
DUF3920
D-ala ligase

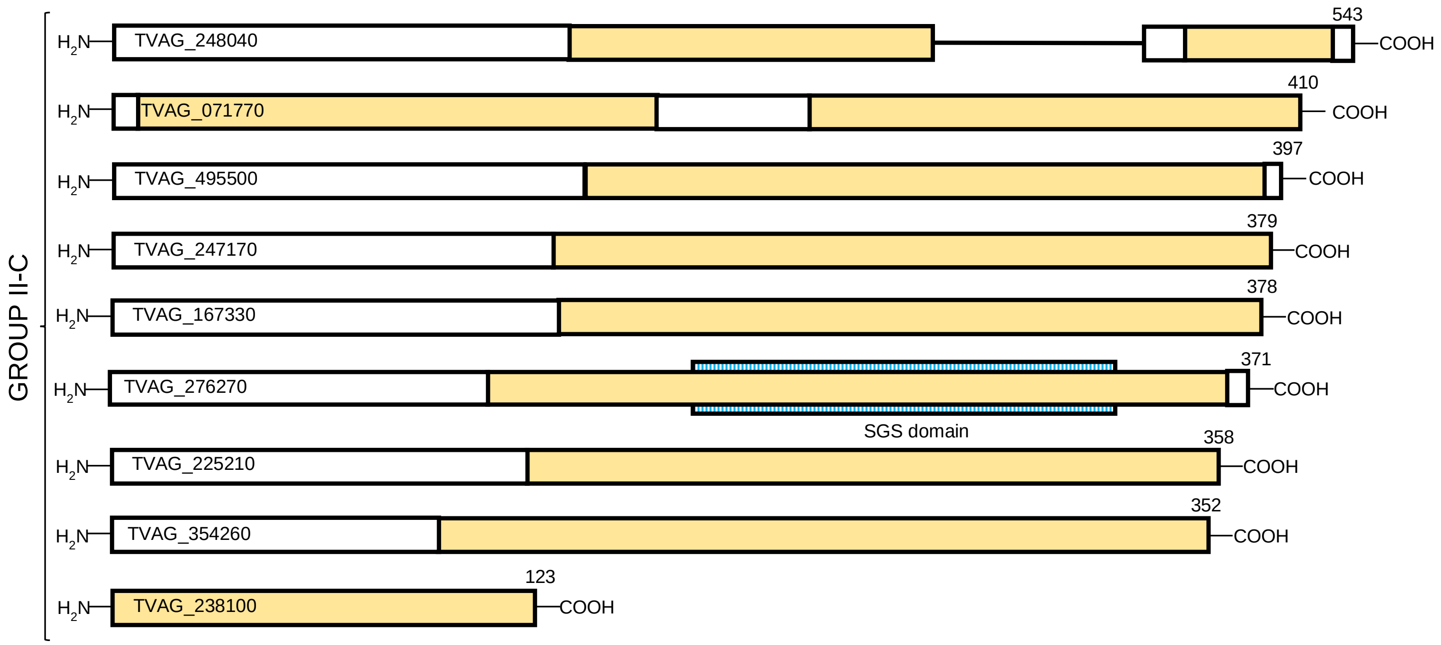

Supplementary Figure S1. *In silico* analysis of the 29 actin-like deduced aa sequences reported in the *T. vaginalis* genome database. Turquoise box: ATP-binding site. Cream box: actin-like ATPase domain. Green box: putative phospholipase D-type domain. Red dotted box: leucine zipper domain. Specific domains are indicated in certain actin-like protein fragments. Groups are based on Supplementary Table S1 description.
