# Supplementary material for: Omics Analyses of Trichomonas vaginalis Actin and Tubulin and Their Participation in Intercellular Interactions and Cytokinesis
Source: Genes (Basel). 2022 Jun 15;13(6):1067. doi: 10.3390/genes13061067 (PMC9222396; doi:10.3390/genes13061067)
Supplement: Supplementary file 1 [file genes-13-01067-s001.zip › sentGalley Suppl. FIGURES-100622/Supplementary Figure S11.pdf]

A

```
TvTUBα1 -MREVISIHVGQAGVQIGNGCWELYCLEHGIQPDGQLPSDKTIGVEDDAFNTFFSETGAG 59
TvTUBβ2 MVREIVHIQAGQCGNQIGAKFWEVISDEHGIDPTGSYHGDSDLQL--ERINVYYNEATGG 58
         :**:: *:.**.* ***   **: . ****:* *.   .*. : :  : :*.::.*: .*

TvTUBα1 KHVPRAVFVDLEPTVVDEVRTGTYRQLFHPEQLISGKEDAANNYARGHYTVGKEIIDLTL 119
TvTUBβ2 KYVPRAILVDLEPGTMDSVRAGQFGQLFRPDNFVFGQSGAGNNWAKGYYTEGQELCESIL 118
        *:****::*****  .:*.**:*  : ***:*:::: *:..*.**:*:*:** *:*: :  *

                                   2.728
TvTUBα1 DRIRKLADQCTGLQGFLIFHSFGGGTGAGFGSLLLERLSVDYGKKSKLEFTVYPSPQVST 179
TvTUBβ2 DVIRKEAESCDCLQGFQLVHSLGGGTGAGLGTLLLNKLREEYPDRILSTYSIVPSPKVSD 178
        * *** *:.*  **** :.**:*******:*:***::*  :* .:    ::: ***:**

TvTUBα1 AIVEPYNSILATHSMIDHSDCAFMVDNEALYDLCRRALDIERPTYTNLNRLIGQVVSSLT 239
TvTUBβ2 TVVEPYNCTLSVHQLVESADEVFCIDNEALYDICFRTLKLTTPTYGDLNHLVSMVMSGTT 238
        ::*****. *:.*.::: :* .* :*******:* *:*.:  *** :**:*:. *:*. *

TvTUBα1 ASLRFDGALNVDFTEFQTNLVPYARIHFPICSYAPVISAEKAYHEQLSVAEITNSLFEPA 299
TvTUBβ2 CALRFPGQLNSDLRKLAVNLVPFPRLHFFIVGFAPLTSRGSQQYRALTVPELTSQLFDNK 298
        .:*** * ** *: :: .****: *:** * .:**: *  .  :. *:* *:*..**:

             1.2380
TvTUBα1 NMMVKCDPRHGKYMACTLLYRGDVVPKDVSAAVATIKTKRTIQFVDWCPTGFKMGINYQP 359
TvTUBβ2 NMMAACDPRRGVYLTASAHFRGRMSSKEVDEQMLNIQARNTSYFVEWIPSNVKSAICDIP 358
        ***. ****:* *::.:  :** :  *:*.  : .*:::.*  **:* *:..* .*   *

TvTUBα1 PTVVPGGDLAKVQRAVCMLANTTAIAEAWSRLDHKFDLMYAKRAFVHWYVGEGMEEAEFP 419
TvTUBβ2 PRGL--------KMAATFIGNTTAFRELFTRVDSQFQKMYARRAFIHWYVNEGLETVEFD 410
        *  :         : *. ::.****: *  ::*:* :*: ***:***:****.**:* .**

TvTUBα1 EAREDLALLEKDYDEVAAESVEGG--DEEEDGGEM-- 452
TvTUBβ2 EARSNMTDLIQEYEMYETAGVEEQGEGEEDEEGEAAA 447
        ***.::: * ::*:   : .**    .**:: **
```

B

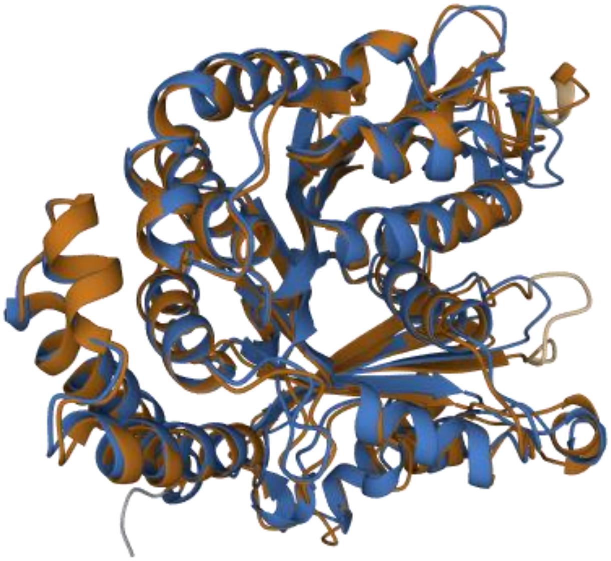

Supplementary Figure S11. Alignment and modeling of TvTUBα1 and TvTUBβ2 identified by LC-ESI-MS. **A**) Peptides identified of the TvTUBα1 are shaded in brown and for TvTUBβ2 are shaded in blue. The aa box are putative antigen regions common among the two proteins. The top number

indicates the overall prediction for the antigen (threshold for this model is 0.5, VaxiJen v2.0). **B**) Comparison of 3D structure of the TvTUBα1 (brown) and TvTUBβ2 (blue). The overlapping of TvTUBα1 against TvTUBβ2 structures was made in PDB Pairwise Structure Alignment (https://www.rcsb.org/alignment). TM-score: 0.95, SI%: 43 and SS%: 62. **TM-score**, template modeling score. It is a measure of topological similarity between the template and 3D model structures. The TM-score ranges between 0 and 1, where 1 indicates a perfect match and 0 is no match between the two structures. **SI%** (sequence identity percentage) is the percent of paired residues in the alignment that are identical in sequence. **SS%** (sequence similarity percentage) is the percent of paired residues in the alignment that are similar in sequence (Xu and Zhang, 2010).
* Represents identical nucleotides.
