# Supplementary material for: Omics Analyses of Trichomonas vaginalis Actin and Tubulin and Their Participation in Intercellular Interactions and Cytokinesis
Source: Genes (Basel). 2022 Jun 15;13(6):1067. doi: 10.3390/genes13061067 (PMC9222396; doi:10.3390/genes13061067)
Supplement: Supplementary file 1 [file genes-13-01067-s001.zip › sentGalley Suppl. FIGURES-100622/Supplementary Figure S2.pdf]

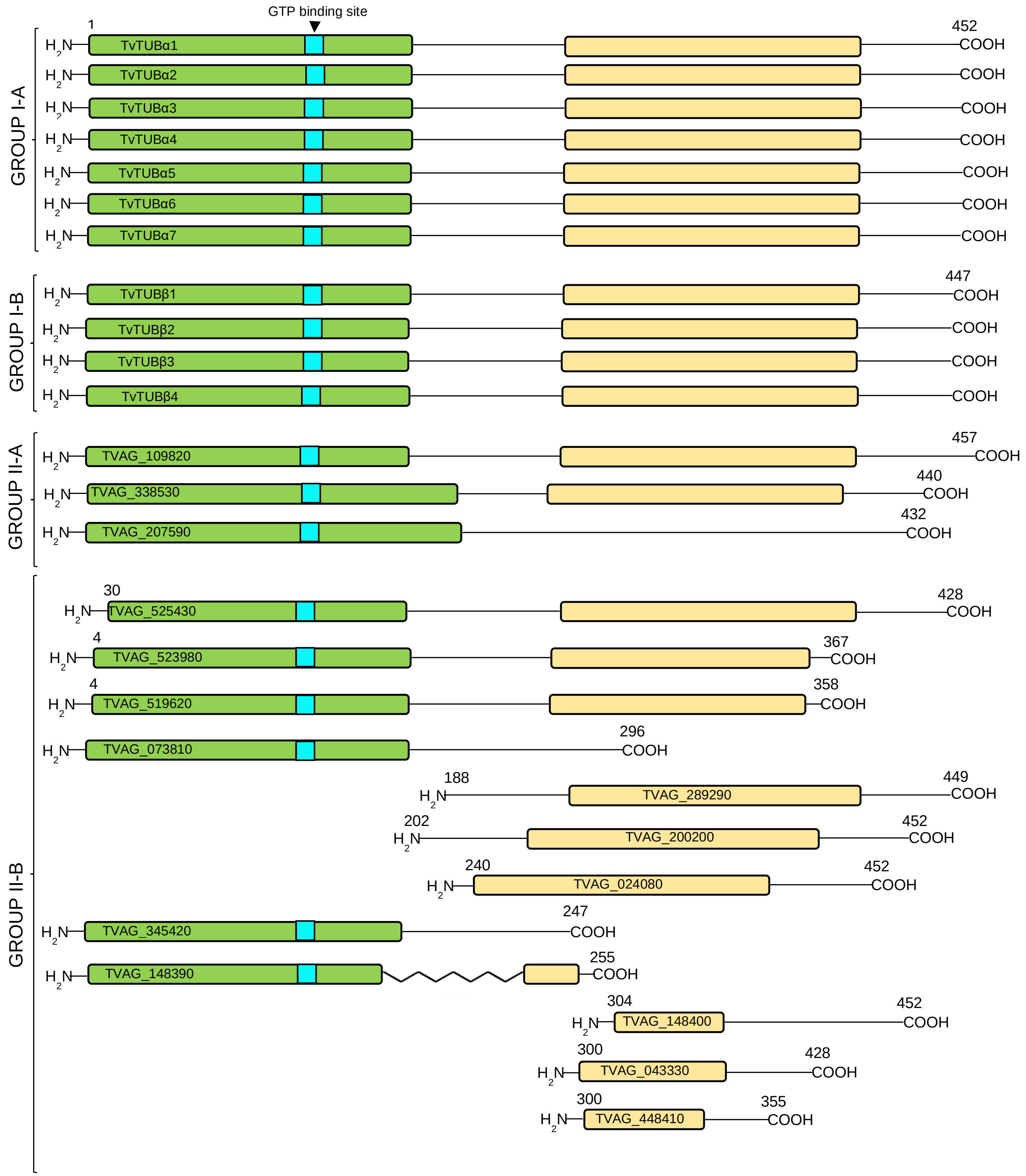

GTP binding site
GROUP I-A
1
452
TvTUBα1
TvTUBα2
TvTUBα3
TvTUBα4
TvTUBα5
TvTUBα6
TvTUBα7
GROUP I-B
447
TvTUBβ1
TvTUBβ2
TvTUBβ3
TvTUBβ4
GROUP II-A
457
TVAG_109820
440
TVAG_338530
432
TVAG_207590
GROUP II-B
30
428
TVAG_525430
4
367
TVAG_523980
4
358
TVAG_519620
296
TVAG_073810
188
449
TVAG_289290
202
452
TVAG_200200
240
452
TVAG_024080
247
TVAG_345420
255
TVAG_148390
304
452
TVAG_148400
300
428
TVAG_043330
300
355
TVAG_448410
H_2N
COOH

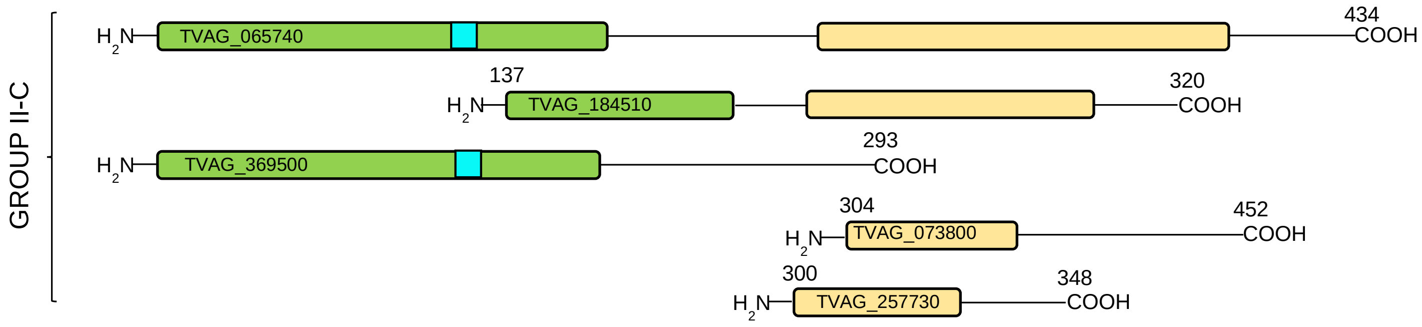

Supplementary Figure S2. *In silico* analysis of the 31 tubulin-like deduced aa sequences reported in the *T. vaginalis* genome database. The green box: tubulin GTPase domain. The turquoise box: GTP-binding site. The cream box: C-terminal tubulin domain. Groups are based on Supplementary Table S1 description.
