# Supplementary material for: Omics Analyses of Trichomonas vaginalis Actin and Tubulin and Their Participation in Intercellular Interactions and Cytokinesis
Source: Genes (Basel). 2022 Jun 15;13(6):1067. doi: 10.3390/genes13061067 (PMC9222396; doi:10.3390/genes13061067)
Supplement: Supplementary file 1 [file genes-13-01067-s001.zip › sentGalley Suppl. FIGURES-100622/Supplementary Figure S3.pdf]

```
CNCD147   ATGGCTAAAGAAGACGTTCAGACACTCGTTATCGATAACGGCTCTGGTATGTGCAAGGCTGGCTTCTCTGGTGATGAAGCCCCACG 86
G3        ATGGCTGAAGAAGACGTTCAGACACTCGTTATCGATAACGGCTCTGGTATGTGCAAGGCTGGCTTCTCTGGTGATGAAGCCCCACG 86
          ******.*******************************************************************************

CNCD147   CTCTGTCTTCCCATCCGTTGTCGGCCGTCCAAAGTACAAACAACAATTAGTTGGTGGCAACGCCAAGGATGTCTTCGTTGGTGATG 172
G3        CTCTGTTTTCCCATCCGTTGTCGGCCGTCCAAAGTACAAACAACAATTAGTTGGTGGCAATGCCAAGGACGTTTTCGTTGGTGATG 172
          ****** ***************************************************** ******** ** *************

CNCD147   AAGCTTGCTCCAAGGCTGGTGTCCTCATCCTCAAGTACCCAATTGAACACGGTATTGTCAACAACTGGGATGATATGGAAAAGATC 258
G3        AAGCCTGCTCCAAGGCTGGTGTCCTCATCCTCAAGTACCCAATTGAACACGGTATCGTCAACAACTGGGATGATATGGAAAAGATC 258
          **** ************************************************** ******************************

CNCD147   TGGCACCACACATTCTACAACGAACTTCGTGTTGACCCAACAGAGCACCCAGTTCTTCTTACAGAAGCCCCACTCAACCCAAAGGC 344
G3        TGGCACCACACATTCTACAACGAACTTCGTGTTGACCCAACAGAGCACCCAGTTCTTCTTACAGAAGCCCCACTCAACCCAAAGGC 344
          **************************************************************************************

CNCD147   TAACCGTGAGAAAATGATCTCCCTCATGTTCGACACATTCAACGTCCCATCATTCTATGTCGGCATCCAAGCCGTTCTTTCCCTCT 430
G3        TAACCGTGAGAAAATGATCTCCCTCATGTTCGACACATTCAACGTCCCATCATTCTATGTCGGCATCCAAGCCGTTCTTTCCCTCT 430
          **************************************************************************************

CNCD147   ACTCCTCTGGCCGTACAACAGGTATCGTTTTCGATGCTGGTGATGGTGTTTCCCACACAGTTCCAATTTACGAAGGCTACTCCCTT 516
G3        ACTCCTCTGGCCGTACAACAGGTATCGTTTTCGATGCTGGTGATGGTGTTTCCCACACAGTTCCAATTTACGAAGGCTACTCCCTT 516
          **************************************************************************************

CNCD147   CCACACGCCATCATGAGACTTAACCTCGCTGGCCGTGATCTCACAGCCTGGATGGTCAAGCTTCTCACAGAGCGTGGCAATGCTTT 602
G3        CCACACGCCATCATGAGACTTAACCTCGCTGGCCGTGATCTCACAGCCTGGATGGTCAAGCTTCTCACAGAGCGTGGCAATGCTTT 602
          **************************************************************************************

CNCD147   CAACACAACAGCCGAAAAGGAAATCGTTCGTGACATCAAGGAGAAGCTTTGCTATGTTGCCCTCGACTTCGATGCTGAAATGGAGA 688
G3        CAACACAACAGCCGAAAAGGAAATCGTTCGTGACATCAAGGAGAAGCTTTGCTATGTCGCCCTCGACTTCGATGCTGAAATGGAGA 688
          ********************************************************* ****************************

CNCD147   AGGCCGCTACAGACTCCTCCATCAACGTCAACTACACACTTCCAGATGGCAACGTCATCACAATCGGCAATGAGCGCTTCCGCTGC 774
G3        AGGCCGCTACAGACTCCTCCATCAACGTCAACTACACACTTCCAGATGGCAACGTCATCACAATCGGCAATGAGCGCTTCCGCTGC 774
          **************************************************************************************

CNCD147   CCAGAAATGCTCTTCAAGCCATACTTCGATGGTATGGAATACGATGGTATCGACAAGACACTCTTCGACTCCATCATGAAGTGCGA 860
G3        CCAGAAATGCTCTTCAAGCCATACTTCGATGGTATGGAATACGATGGTATCGACAAGACACTCTTCGACTCCATCATGAAGTGCGA 860
          **************************************************************************************

CNCD147   TATCGATGTTCGTAAGGATCTCTACGCTAACATCGTTCTTTCTGGTGGCACAACAATGTTCAACGGCATCGCCGAACGTCTTGACA 946
G3        TATCGATGTTCGTAAGGATCTCTACGCTAACATCGTTCTTTCTGGTGGCACAACAATGTTCCAGGGCATCGCCGAACGTCTTGACA 946
          ************************************************************* * **********************

CNCD147   AGGAAATCACCGCTCTTGCTCCACCAACAATGAAGGTCAAGATCGTCGCCCCAGAAGAGCGTAAGTACGCCGTTTGGGTCGGTGGC 1032
G3        AGGAAATCACAGCTCTTGCTCCACCAACAATGAAGGTCAAGATCGTCGCCCCAGAAGAGCGTAAGTACGCCGTTTGGGTCGGTGGC 1032
          ********** ***************************************************************************

CNCD147   TCCATCCTTGCTTCCCTCGCTACATTCCCACAGATGGTTATCACCAAGGAGGAATACGACGAGGCTGGCCCATCCATCGTTCACCG 1118
G3        TCCATCCTTGCTTCCCTCGCTACATTCCCACAGATGGTTATCACCAAGGAGGAATACGACGAGGCTGGCCCATCCATCGTTCACCG 1118
          **************************************************************************************

CNCD147   CAAGTGCTTCTAA 1131
G3        TAAGTGCTTCTAA 1131
           ************
```

Supplementary Figure S3. Nucleotide sequence alignment between the *tvact1* gene amplified from the *T. vaginalis* CNCD147 isolate and G3 from the *T. vaginalis* genome. The letters in red indicate the nt changes found after the amplicon DNA sequencing. * Represents identical nucleotides.
