# Supplementary material for: Omics Analyses of Trichomonas vaginalis Actin and Tubulin and Their Participation in Intercellular Interactions and Cytokinesis
Source: Genes (Basel). 2022 Jun 15;13(6):1067. doi: 10.3390/genes13061067 (PMC9222396; doi:10.3390/genes13061067)
Supplement: Supplementary file 1 [file genes-13-01067-s001.zip › sentGalley Suppl. FIGURES-100622/Supplementary Figure S5.pdf]

```
CNCD147   MAKEDVQTLVIDNGSGMCKAGFSGDEAPRSVFPSVVGRPKYKQQLVGGNAKDVFVGDEAC 60
TvACT1    MAEEDVQTLVIDNGSGMCKAGFSGDEAPRSVFPSVVGRPKYKQQLVGGNAKDVFVGDEAC 60
          ** *********************************************************

CNCD147   SKAGVLILKYPIEHGIVNNWDDMEKIWHHTFYNELRVDPTEHPVLLTEAPLNPKANREKM 120
TvACT1    SKAGVLILKYPIEHGIVNNWDDMEKIWHHTFYNELRVDPTEHPVLLTEAPLNPKANREKM 120
          ************************************************************

CNCD147   ISLMFDTFNVPSFYVGIQAVLSLYSSGRTTGIVFDAGDGVSHTVPIYEGYSLPHAIMRLN 180
TvACT1    ISLMFDTFNVPSFYVGIQAVLSLYSSGRTTGIVFDAGDGVSHTVPIYEGYSLPHAIMRLN 180
          ************************************************************

CNCD147   LAGRDLTAWMVKLLTERGNAFNTTAEKEIVRDIKEKLCYVALDFDAEMEKAATDSSINVN 240
TvACT1    LAGRDLTAWMVKLLTERGNAFNTTAEKEIVRDIKEKLCYVALDFDAEMEKAATDSSINVN 240
          ************************************************************

CNCD147   YTLPDGNVITIGNERFRCPEMLFKPYFDGMEYDGIDKTLFDSIMKCDIDVRKDLYANIVL 300
TvACT1    YTLPDGNVITIGNERFRCPEMLFKPYFDGMEYDGIDKTLFDSIMKCDIDVRKDLYANIVL 300
          ************************************************************

CNCD147   SGGTTMFNGIAERLDKEITALAPPTMKVKIVAPEERKYAVWVGGSILASLATFPQMVITK 360
TvACT1    SGGTTMFQGIAERLDKEITALAPPTMKVKIVAPEERKYAVWVGGSILASLATFPQMVITK 360
          ******* ****************************************************

CNCD147   EEYDEAGPSIVHRKCF 376
TvACT1    EEYDEAGPSIVHRKCF 376
```

**Supplementary Figure S5.** Amino acid alignment of the TvACT1 protein of the *T. vaginalis* CNCD147 isolate and the G3 *T. vaginalis* genome. The letters in red indicate the aa changes found after amplicon DNA sequencing. * Represents identical amino acid.
