# Supplementary material for: Omics Analyses of Trichomonas vaginalis Actin and Tubulin and Their Participation in Intercellular Interactions and Cytokinesis
Source: Genes (Basel). 2022 Jun 15;13(6):1067. doi: 10.3390/genes13061067 (PMC9222396; doi:10.3390/genes13061067)
Supplement: Supplementary file 1 [file genes-13-01067-s001.zip › sentGalley Suppl. FIGURES-100622/Supplementary Figure S6.pdf]

```
CNCD147  MREVISIHVGQAGVQIGNGCWELYCLEHGIQPDGQLPSDKTIGVEDDAFNTFFSETGAGK 60
G3       MREVISIHVGQAGVQIGNGCWELYCLEHGIQPDGQLPSDKTIGVEDDAFNTFFSETGAGK 60
         ************************************************************

CNCD147  HVPRAVFVDLEPTVVDEVRTGTYRQLFHPEQLISGKEDAANNYARGHYTVGKEIIDLTLD 120
G3       HVPRAVFVDLEPTVVDEVRTGTYRQLFHPEQLISGKEDAANNYARGHYTVGKEIIDLTLD 120
         ************************************************************

CNCD147  RIRKLADQCTGLQGFLIFHSFGGGTGAGFGSLLLERLSVDYGKKSKLEFTVYPSPQVSTA 180
G3       RIRKLADQCTGLQGFLIFHSFGGGTGAGFGSLLLERLSVDYGKKSKLEFTVYPSPQVSTA 180
         ************************************************************

CNCD147  IVEPYNSILATHSMIDHSDCAFMVDNEALYDLCRRALDIERPTYTNLNRLIGQVVSSLTA 240
G3       IVEPYNSILATHSMIDHSDCAFMVDNEALYDLCRRALDIERPTYTNLNRLIGQVVSSLTA 240
         ************************************************************

CNCD147  SLRFDGALNVDFTEFQTNLVPYARIHFPICSYAPVISAEKAYHEQLSVAEITNSLFEPAN 300
G3       SLRFDGALNVDFTEFQTNLVPYARIHFPICSYAPVISAEKAYHEQLSVAEITNSLFEPAN 300
         ************************************************************

CNCD147  MMVKCDPRHGKYMACTLLYRGDVVPKDVSAAVATIKTKRTIQFVDWCPTGFKMGINYQPP 360
G3       MMVKCDPRHGKYMACTLLYRGDVVPKDVSAAVATIKTKRTIQFVDWCPTGFKMGINYQPP 360
         ************************************************************

CNCD147  TVVPGGDLAKVQRAVCMLANTTAIAEAWSRLDHKFDLMYAKRAFVHWYVGEGMEEAEFPE 420
G3       TVVPGGDLAKVQRAVCMLANTTAIAEAWSRLDHKFDLMYAKRAFVHWYVGEGMEEAEFPE 420
         ************************************************************

CNCD147  AREDLALLEKDYDEVAAESVEGGDEEEDGGEM 452
G3       AREDLALLEKDYDEVAAESVEGGDEEEDGGEM 452
         ********************************
```

Supplementary Figure S6. Amino acid alignment of the TvTUBα1 protein of the *T. vaginalis* CNCD147 isolate and the G3 *T. vaginalis* genome. * Represents identical amino acid.
