# Supplementary material for: Omics Analyses of Trichomonas vaginalis Actin and Tubulin and Their Participation in Intercellular Interactions and Cytokinesis
Source: Genes (Basel). 2022 Jun 15;13(6):1067. doi: 10.3390/genes13061067 (PMC9222396; doi:10.3390/genes13061067)
Supplement: Supplementary file 1 [file genes-13-01067-s001.zip › sentGalley Suppl. FIGURES-100622/Supplementary Figure S7.pdf]

MAEEDVQTLVIDNGSGMCKAGFSGDEAPRSVFPSVVGRPKYKQQLVGGNAKDVFVGDEAC

SKAGVLILKYPIEHGIVNNWDDMEKIWHHTFYNELRVDPTEHPVLLTEAPLNPKANREKM

ISLMFDTFNVPSFYVGIQAVLSLYSSGRTTGIVFDAGDGVSHTVPIYEGYSLPHAIMRLN

LAGRDLTAWMVKLLTERGNAFNTTAEKEIVRDIKEKLCYVALDFDAEMEKAATDSSINVN

YTLPDGNVITIGNERFRCPEMLFKPYFDGMEYDGIDKTLFDSIMKCDIDVRKDLYANIVL

SGGTTMFQGIAERLDKEITALAPPTMKVKIVAPEERKYAVWVGGSILASLATFPQMVITK

EEYDEAGPSIVHRKCF

Supplementary Figure S7. Localization of peptides identified by LC-ESI-MS of the spots 1-3 of actin protein (TvACT4, TVAG_249200). The aa shaded in gray correspond to the common peptides identified in the three spots. The aa in the black boxes are the differential peptides identified only in spot 1; the underlined aa correspond to the differential peptides identified only in spot 2: The grey boxes correspond to the aa of the differential peptides identified only in spot 3.
