# Supplementary material for: Omics Analyses of Trichomonas vaginalis Actin and Tubulin and Their Participation in Intercellular Interactions and Cytokinesis
Source: Genes (Basel). 2022 Jun 15;13(6):1067. doi: 10.3390/genes13061067 (PMC9222396; doi:10.3390/genes13061067)
Supplement: Supplementary file 1 [file genes-13-01067-s001.zip › sentGalley Suppl. FIGURES-100622/Supplementary Figure S8.pdf]

MVREIVHIQAGQCGNQIGAKFWEVISDEHGIDPTGSYHGDSDLQLERINVYYNEATGGKY
VPRAILVDLEPGTMDSVRAGQFGQLFRPDNFVFGQSGAGNNWAKGYYTEGQELCESILDV
IRKEAESCDCLQGFQLVHSLGGGTGAGLGTLLLNKLREEYPDRILSTYSIVPSPKVSDTV
VEPYNCTLSVHQLVESADEVFCIDNEALYDICFRTLKLTTPTYGDLNHLVSMVMSGTTCA
LRFPGQLNSDLRKLAVNLVPFPRLHFFIVGFAPLTSRGSQQYRALTVPELTSQLFDNKNM
MAACDPRRGVYLTASAHFRGRMSSKEVDEQMLNIQARNTSYFVEWIPSNVKSAICDIPPR
GLKMAATFIGNTTAFRELFTRVDSQFQKMYARRAFIHWYVNEGLETVEFDEARSNMTDLI
QEYEMYETAGVEEQGEGEEDEEGEAAA

Supplementary Figure S8. Localization of peptides identified by LC-ESI-MS of the spots 1 and 3 of tubulin protein (TvTUBβ2; TVAG_456920). The black box indicates the common peptides identified in the two spots. Shaded in grey indicates the differential peptides identified only in spot 3.
