# Supplementary material for: Omics Analyses of Trichomonas vaginalis Actin and Tubulin and Their Participation in Intercellular Interactions and Cytokinesis
Source: Genes (Basel). 2022 Jun 15;13(6):1067. doi: 10.3390/genes13061067 (PMC9222396; doi:10.3390/genes13061067)
Supplement: Supplementary file 1 [file genes-13-01067-s001.zip › sentGalley Suppl. FIGURES-100622/Supplementary Figure S9.pdf]

MREVISIHVGQAGVQIGNGCWELYCLEHGIQPDGQLPSDKTIGVEDDAFNTFFSETGAGK
HVPRAVFVDLEPTVVDEVRTGTYRQLFHPEQLISGKEDAANNYARGHYTVGKEIIDLTLD
RIRKLADQCTGLQGFLIFHSFGGGTGAGFGSLLLERLSVDYGKKSKLEFTVYPSPQVSTA
IVEPYNSILATHSMIDHSDCAFMVDNEALYDLCRRALDIERPTYTNLNRLIGQVVSSLTA
SLRFDGALNVDFTEFQTNLVPYARIHFPICSYAPVISAEKAYHEQLSVAEITNSLFEPAN
MMVKCDPRHGKYMACTLLYRGDVVPKDVSAAVATIKTKRTIQFVDWCPTGFKMGINYQPP
TVVPGGDLAKVQRAVCMLANTTAIAEAWSRLDHKFDLMYAKRAFVHWYVGEGMEEAEFPE
AREDLALLEKDYDEVAAESVEGGDEEEDGGEM

Supplementary Figure S9. Localization of peptides identified by LC-ESI-MS of the spot 2 and 3 of tubulin protein (TvTUBα1; TVAG_359090). The black box indicates the peptides identified in both spots.
