# Supplementary material for: Omics Analyses of Trichomonas vaginalis Actin and Tubulin and Their Participation in Intercellular Interactions and Cytokinesis
Source: Genes (Basel). 2022 Jun 15;13(6):1067. doi: 10.3390/genes13061067 (PMC9222396; doi:10.3390/genes13061067)
Supplement: Supplementary file 1 [file genes-13-01067-s001.zip › sentGalley Suppl. TABLES-100622/Supplementary Table S1.pdf]

Supplementary Table S1. Analysis of DNA sequences encoding actin-like protein segments found in the *T. vaginalis* genome database.

PROTEIN			TVAG_	CONTIG	ORF	aa	*pI*	MW	αACT Homo sapiens (HuACT)				ESTs
									Identity		Structure		
Group		Name							Gene (%)	Protein (%)	Identity (%)	TM-score	
I		TvACT1	160060	DS113250	1128	376	5.02	41.84	69.56	74.27	75	0.97	168
		TvACT2	172680	DS113193	1128	376	5.02	41.84	70.03	74.27	75	0.97	503
		TvACT3	200190	DS114265	1128	376	5.02	41.84	69.83	74.27	74	0.96	322
		TvACT4	249200	DS113187	1128	376	5.02	41.84	69.56	74.27	75	0.97	404
		TvACT5	310030	DS113416	1128	376	5.02	41.84	69.11	74.27	75	0.97	239
		TvACT6	485210	DS113547	1128	376	5.02	41.84	69.83	74.27	75	0.97	73
		TvACT7	149090	DS113750	1128	376	5.02	41.84	69.91	74.27	75	0.97	141
		TvACT8	054030	DS114248	1128	376	5.12	41.82	69.56	74.01	74	0.97	338
		TvACT9	090470	DS113682	1128	376	5.05	41.80	69.65	74.27	75	0.97	231
		TvACT10	337240	DS113974	1128	376	5.12	41.84	69.77	74.27	75	0.97	181
II	A		512800	DS139403	1098	366	5.24	40.72	67.98	72.95	75	0.96	86
			150270	DS113237	1041	347	5.02	38.59	63.77	63.61	74	0.91	140
			534990	DS116858	714	238	5.02	26.46	44.71	48.01	75	0.42	75
	B		371880	DS113281	1260	420	6.04	46.64	41.58	31.60	36	0.83	46
			043970	DS113279	1227	409	6.19	46.40	36.88	22.43	25	0.80	1
			094140	DS113185	1191	397	5.61	44.25	53.81	39.95	41	0.91	0
			189860	DS113211	1116	372	4.62	41.43	34.94	21.38	25	0.85	4
			027630	DS113305	1095	365	5.52	40.38	51.40	45.50	48	0.94	0
			161530	DS113499	1080	360	5.12	39.00	42.40	35.98	38	0.93	4
			434970	DS114152	918	306	6.05	33.75	34.60	20.57	26	0.79	33
	C		248040	DS113963	1629	543	5.23	60.26	31.48	26.64	37	0.82	0
			071770	DS113178	1230	410	5.81	46.21	39.71	23.32	26	0.81	0
			495500	DS113254	1191	397	5.00	44.48	37.57	20.62	22	0.83	2
			247170	DS113212	1137	379	5.44	41.98	52.47	48.16	50	0.97	3
			167330	DS113191	1134	378	5.85	42.51	43.61	30.75	33	0.96	1
			276270	DS113355	1113	371	5.21	41.62	50.04	41.42	43	0.96	0
			225210	DS113224	1074	358	4.89	39.84	43.37	33.95	35	0.94	3
			354260	DS113673	1056	352	5.50	39.14	34.60	20.30	23	0.80	11
			238100	DS113188	369	123	4.86	14.37	18.84	16.98	57	0.29	1

There are 29 DNA segments encoding for actin-like protein fragments in the *Tv* genome located in different contigs. Ten genes show high identity at the nucleotide and amino acid level with HuACT (Group I) and are considered bonafide actins Three are gene fragments encoding protein fragments with high homology to Group I (Group II-A) and seven with divergence to Group I (Group II-B), both with evidence of expression in the different proteomes analyzed (Supplementary Table S3). Nine fragments also with divergence to Group I proteins without evidence of expression (Group II-C). Most of them are expressed at the mRNA level, as confirmed by the presence of ESTs. **ORF**, Open Reading Frame; **bp**, Base Pairs; **aa**, Amino Acids; ***pI***, Isoelectric Point; **MW**, Molecular Weight; **ESTs**, Expressed Sequence Tags. **TM-score**, template modeling score. It is a measure of topological similarity between the template and 3D model structures (Xu and Zhang, 2010). The TM-score ranges between 0 and 1, where 1 indicates a perfect match and 0 is no match between the two structures. See Suppl. Table S9 for more information on the predicted 3D structure of each aa sequence.
