# Supplementary material for: Omics Analyses of Trichomonas vaginalis Actin and Tubulin and Their Participation in Intercellular Interactions and Cytokinesis
Source: Genes (Basel). 2022 Jun 15;13(6):1067. doi: 10.3390/genes13061067 (PMC9222396; doi:10.3390/genes13061067)
Supplement: Supplementary file 1 [file genes-13-01067-s001.zip › sentGalley Suppl. TABLES-100622/Supplementary Table S10.pdf]

Supplementary Table S10. Predicted 3D structures of the tubulin-like deduced aa sequences found in the *T. vaginalis* genome database.

Protein				Template	Sequence Identity (%)	Description	GMQE	Ramachandran Favoured (%)	3D Model
Group		Name	TVAG_						
I	A	TvTUBα1	359090	6u42.62	84.82	Tubulin alpha	0.84	97.00	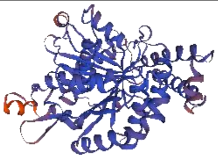
		TvTUBα2	196270	6u42.62	84.82	Tubulin alpha	0.84	97.00	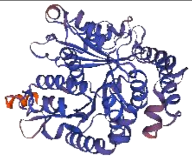
		TvTUBα3	206890	6u42.62	84.82	Tubulin alpha	0.84	97.00	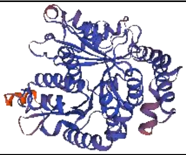
		TvTUBα4	312330	6u42.62	84.82	Tubulin alpha	0.84	97.00	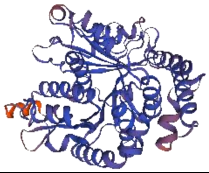
		TvTUBα5	360890	6u42.62	84.82	Tubulin alpha	0.84	97.00	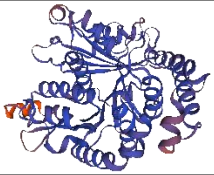
		TvTUBα6	448390	6u42.62	84.41	Tubulin alpha	0.85	97.00	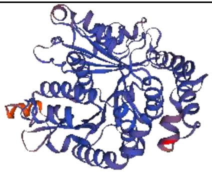
		TvTUBα7	467840	6u42.62	84.41	Tubulin alpha	0.85	97.00	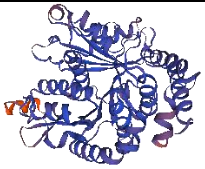
	B	TvTUBβ1	034440	6u42.5	77.83	Tubulin beta	0.84	96.98	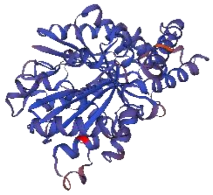
		TvTUBβ2	456920	6u42.5	78.11	Tubulin beta	0.84	97.22	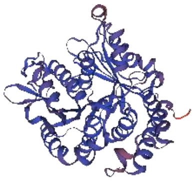
		TvTUBβ3	008680	6u42.5	78.11	Tubulin beta	0.84	97.22	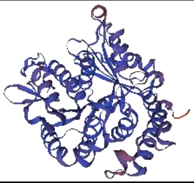

		TvTUBβ4	062880	6u42.5	78.11	Tubulin beta	0.84	97.22	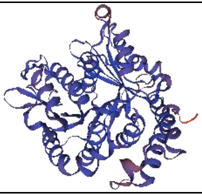
II	A		109820	7qj0.1	53.08	Tubulin gamma-1 chain	0.73	90.00	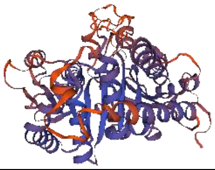
			338530	3jaw.1	70.34	Tubulin beta chain	0.84	96.96	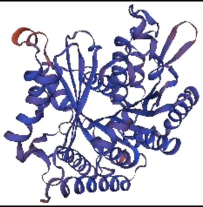
			207590	5mjs.1	25.79	Tubulin beta chain	0.54	92.29	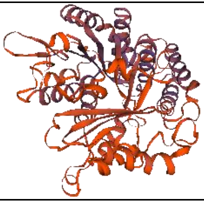
	B		525430	7rro.28	79.29	Tubulin beta-4B chain	0.85	97.72	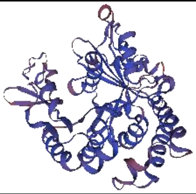
			523980	6u42.32	84.66	Tubulin alpha	0.84	96.35	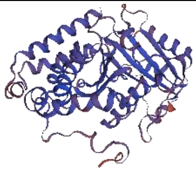
			519620	6u42.62	85.75	Tubulin alpha	0.86	96.07	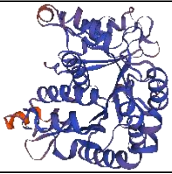
			073810	6u42.15	79.35	Tubulin beta	0.81	96.76	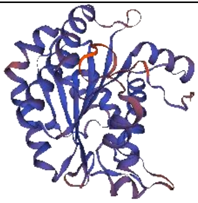
			289290	3jaw.1	64.40	Tubulin beta chain	0.81	98.76	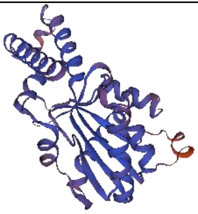

			200200	7rro.26	61.34	Tubulin beta-4B chain	0.76	94.09	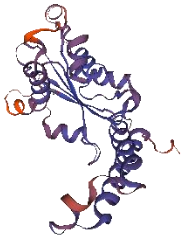
			024080	7miz.51	70.53	Tubulin alpha chain	0.73	92.78	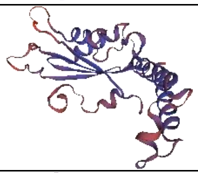
			345420	6u42.12	84.36	Tubulin alpha	0.85	94.23	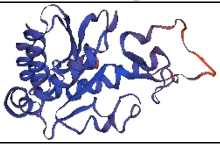
			148390	7rro.24	86.80	Tubulin beta-4B chain	0.86	96.41	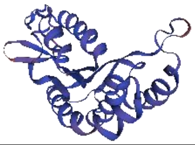
			148400	7rro.26	65.73	Tubulin beta-4B chain	0.77	95.56	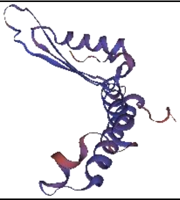
			043330	7n32.1	75.00	Tubulin alpha chain	0.71	95.24	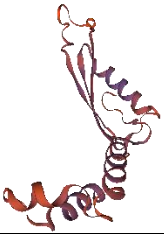
			448410	5sye.3	90.57	Tubulin alpha-chain	0.61	94.12	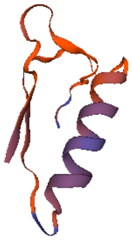
	C		065740	7n6g.105	29.90	Tubulin beta	0.67	92.89	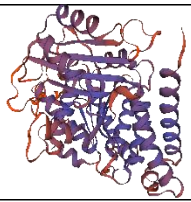
			184510	7rro.22	44.04	Tubulin beta-4B chain	0.74	94.28	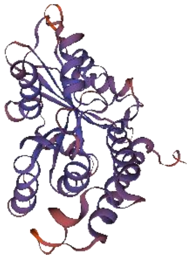

			369500	7as4.1	53.28	Tubulin gamma-1 chain	0.72	91.10	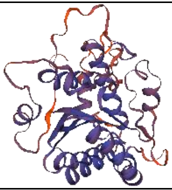
			73800	7rro.26	65.73	Tubulin beta-4B chain	0.77	95.56	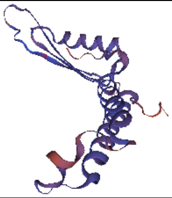
			257730	7n32.1	78.72	Tubulin alpha chain	0.64	95.56	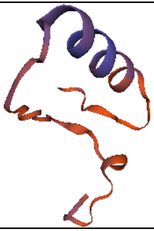

3D Structure prediction for each tubulin-like protein fragment found in the *Tv* genome database was obtained in the Swiss Model Server from EXPASY (https://swissmodel.expasy.org/) [Waterhouse, A., Bertoni, M., Bienert, S., Studer, G., Tauriello, G., Gumienny, R., Heer, F.T., de Beer, T.A.P., Rempfer, C., Bordoli, L., Lepore, R. and Schwede, T. 2018. SWISS-MODEL: homology modelling of protein structures and complexes. Nucleic Acids Res. 46, W296-W303; Xu J, and Zhang Y. 2010. How significant is a protein structure similarity with TM-score = 0.5? Bioinformatics. 26(7):889-95] using the deduced aa sequence. These data were one of the parameters to consider for the identification of possible alpha (Group I-A) and beta tubulins (Group I-B) in *Tv*. **GMQE**, Global Model Quality Estimate. It is a quality estimate, which combines properties from the target-template alignment and the template structure. GMQE gives an overall model quality measurement between 0 and 1, with higher numbers indicating higher expected quality (Waterhouse A. *et al*., 2018).
