# Supplementary material for: Omics Analyses of Trichomonas vaginalis Actin and Tubulin and Their Participation in Intercellular Interactions and Cytokinesis
Source: Genes (Basel). 2022 Jun 15;13(6):1067. doi: 10.3390/genes13061067 (PMC9222396; doi:10.3390/genes13061067)
Supplement: Supplementary file 1 [file genes-13-01067-s001.zip › sentGalley Suppl. TABLES-100622/Supplementary Table S2.pdf]

Supplementary Table S2. Analysis of DNA sequences encoding tubulin-like protein segments found in the *T. vaginalis* genome database.

PROTEIN			TVAG_	CONTIG	ORF	aa	*pI*	MW	TUBβ3 Homo sapiens (HuTUBβ)				TUBα1A Homo sapiens (HuTUBα)				ESTs
									Identity		Structure		Identity		Structure		
Group		Name							Gene (%)	Protein (%)	Identity (%)	TM-score	Gene (%)	Protein (%)	Identity (%)	TM-score	
I	A	TvTUBα1	359090	DS113325	1359	452	4.90	50.11	52.838	38.478	40	0.95	75.423	87.389	89	0.96	187
		TvTUBα2	196270	DS113615	1359	452	4.90	50.11	52.768	38.478	40	0.95	75.497	87.389	89	0.96	140
		TvTUBα3	206890	DS113537	1359	452	4.90	50.11	51.385	38.478	40	0.95	75.865	87.389	89	0.96	191
		TvTUBα4	312330	DS113391	1359	452	4.90	50.11	52.698	38.478	40	0.95	75.570	87.389	89	0.96	182
		TvTUBα5	360870	DS113782	1359	452	4.90	50.11	51.316	38.478	40	0.95	75.644	87.389	89	0.96	122
		TvTUBα6	448390	DS114717	1359	452	4.90	50.12	50.548	38.478	40	0.95	75.276	87.611	89	0.96	5
		TvTUBα7	467840	DS113280	1359	452	4.90	50.12	52.348	38.478	40	0.95	75.791	87.611	89	0.96	41
	B	TvTUBβ1	034440	DS113428	1344	447	4.82	50.03	66.375	75.831	78	0.97	49.577	41.575	43	0.95	21
		TvTUBβ2	456920	DS113186	1344	447	4.82	49.95	68.062	76.053	78	0.97	49.022	41.575	43	0.95	66
		TvTUBβ3	008680	DS113653	1344	447	4.82	49.95	68.209	76.053	78	0.97	49.084	41.575	43	0.95	81
		TvTUBβ4	062880	DS113216	1344	447	4.82	49.95	67.988	76.053	78	0.97	49.894	41.575	43	0.95	113
II	A		109820	DS113339	1374	457	5.64	50.67	38.466	29.536	32	0.87	39.961	25.957	28	0.86	0
			338530	DS113922	1323	440	4.83	49.52	57.594	67.184	71	0.99	47.303	42.035	44	0.96	0
			207590	DS113587	1299	432	5.89	48.92	38.406	22.601	25	0.94	32.862	19.543	85	0.11	0
	B		525430	DS133806	1197	398	5.74	44.64	61.123	67.111	76	0.92	44.610	37.251	43	0.88	25
			523980	DS139360	1104	367	5.35	40.91	43.352	31.004	40	0.80	61.504	71.397	88	0.82	77
			519620	DS141851	1077	358	5.56	39.60	43.497	32.301	40	0.81	60.398	70.732	89	0.80	66
			073810	DS113366	891	296	5.46	32.76	46.721	47.807	79	0.64	33.760	26.362	45	0.61	50
			289290	DS114116	765	254	4.98	28.84	31.845	35.333	65	0.56	27.094	22.838	41	0.55	0
			200200	DS114265	753	250	5.67	28.22	37.132	33.333	61	0.51	27.544	19.079	34	0.51	10
			024080	DS116663	639	212	5.19	23.84	23.787	13.537	32	0.43	33.855	33.186	73	0.45	20
			345420	DS113513	744	211	4.90	23.18	25.712	20.796	44	0.48	36.209	41.685	89	0.46	48
			148390	DS114446	768	198	4.65	21.66	34.036	37.694	83	0.46	27.088	20.796	46	0.43	15
			148400	DS114446	447	148	4.63	16.93	21.045	21.111	66	0.30	17.249	12.719	40	0.29	10
			043330	DS114339	387	128	5.26	14.39	14.634	9.170	35	0.27	21.313	22.395	79	0.29	0
			448410	DS114717	168	55	9.30	6.18	6.657	4.667	36	0.12	8.923	10.643	91	0.12	0
	C		065740	DS113426	1305	434	5.63	48.45	40.909	27.511	29	0.96	41.219	25.546	28	0.94	3
			184510	DS184510	963	320	4.45	36.12	31.202	30.131	44	0.67	30.027	20.513	31	0.66	1
			369500	DS114576	882	293	4.94	32.12	27.522	22.198	37	0.60	30.490	18.495	33	0.58	0
			073800	DS113366	447	148	4.63	16.93	20.778	21.111	66	0.30	17.176	12.719	40	0.29	7
			257730	DS113609	147	48	9.66	5.48	5.556	4.000	38	0.10	7.743	8.869	85	0.11	1

There are 31 sequences encoding for tubulin-like protein segments in the *T. vaginalis* genome located in different contigs that were classified into two groups according to the identity with human tubulin alpha and beta. Group I represents the amino acid sequences with the highest identity to human tubulin α/β. Seven DNA sequences show high identity at the nucleotide and amino acid level with HuTUBα (Group I-A) and four with HuTUBβ (Group I-B) are considered bonafide tubulin proteins. Group II represents the rest of the sequences that could not be classified within group I. Three sequences showed similarity in size with tubulins of Group I, thus, we classified them into subgroup (II-A) and perhaps could represent another kind of tubulin because we did not analyze the identity with tubulin γ, δ, ε, and others. Twelve sequences were grouped into another subgroup (II –B) and included sequence products found in reported proteomes that could be gene fragments. The last group (II-C) represents hypothetical genes because their amino acid products were not found in reported proteomes. Most of them are expressed at the mRNA level, as confirmed by the presence of ESTs. **ORF**, Open Reading Frame; **bp**, Base Pairs; **aa**, Amino Acids; ***pI***, Isoelectric Point; **MW**, Molecular Weight; **ESTs**, Expressed Sequence Tags; **TM-score**, template modeling score. It is a measure of topological similarity between the template and model structures (Xu and Zhang, 2010). The TM-score ranges between 0 and 1, where 1 indicates a perfect match and 0 is no match between the two structures. See Suppl. Table S10 for more information on the predicted 3D structure of each aa sequence.
