# Supplementary material for: Omics Analyses of Trichomonas vaginalis Actin and Tubulin and Their Participation in Intercellular Interactions and Cytokinesis
Source: Genes (Basel). 2022 Jun 15;13(6):1067. doi: 10.3390/genes13061067 (PMC9222396; doi:10.3390/genes13061067)
Supplement: Supplementary file 1 [file genes-13-01067-s001.zip › sentGalley Suppl. TABLES-100622/Supplementary Table S3.pdf]

Supplementary Table S3. Multiple alignment of the 5’- and 3- regulatory sequences of the bonafide actin-encoding genes of *T. vaginalis*.

Group	Name	TVAG_	
**5´-regulatory regions**			+1
I	TvACT1	160060	CTATCGTTCTGAAAACCCACATATTTTCTAGCAAGTTGCCCGAAAAAATTCAGAGTTGTGATACAGAAATAATT**TCACTTCACA**TTCTCTTCTAATG
	TvACT2	172680	CTTAGATGTCCTGGTTATAAATTTTGAGGACTCTAACCGTTGTCTAAAGAAGTGAAATGATTGGAATGTTAACT**TCATTTCATT**TTCTGGAATG
	TvACT3	200190	TTGACACAAAAAACCCACAATGACATGTGAATGATGCCCTTAAAATTGAGAGCGGATTGAAAAATGCTATGACT**TCACA**TTCTCTTCTAATG
	TvACT4	249200	TCGTTCTGAAAACCCACATGAAAGCAAAGGTATGCCCTATCAAATCAGCATAAAACCGTCATTTTTTGATCAGT**TCACA**TTCTCTTCTAATG
	TvACT5	310030	CCACTAATCCGATAAAAGTGACAGAACAGTTCATCTTGCCCGAAAAATTTCAGGGACATTATCAAAATTATAAT**TCACTTCACA**TTCTCTTCTAATG
	TvACT6	485210	ACTAAATGGGACAAAAGACAGAAGGTTTCCCAAATTGAATGGAAGTGACGTGAGAATATTGCCCATTTCAAAAG**TCACTTCATT**TTTCTGGAATG
	TvACT7	149090	CTTCACTTGTTTCAAACCGTTTTTTATATGAATTAATTTGAGAACTTTACAGGTCATAAGAAACACTTTTTCTT**TCACTTCACA**TTCTCTTCTAATG
	TvACT8	054030	GACGAAATAAACCAAAAGTGACGATCCACTTCATATTGCCCGAAATAATTGAGAGGTTTTGGCAAAAAAGAATG**TCACATCACA**TTCTCTTCTAATG
	TvACT9	090470	TGAGATTCTATCGTTTGAAAAACCCACAAGTCCACTTCACGTTTGCCCTATTTCTGAGGGAACTGAAATTGACT**TCACA**TTTACTTCTCATG
	TvACT10	337240	CCATATTTGAATTCCTCTCAAAATATAAAATCCCTTTCAAAAAGAGATTGAAAGAAGGGCAAATTAAAGTGACT**TCATT**TTTCGTCTAATG
**3´-regulatory regions**			
I	TvACT1	160060	TAAACAATTTCGGTATAATATATTTAAATCAAATCAATTTTTTTT**ATTTTT**CTATCCTAATTTATAACTAATTCATAATACTTTATGGCAATAAAGTTAATA
	TvACT2	172680	TAAACAATTTCGGTATAATATATTTCAAAATTAATTAAC**TTTTTT**CTTTAGTGAACCTGTTTTGGAAATCACTTCATTAATATTGTGTTTTAATGGTTTATA
	TvACT3	200190	TAAAAAGTTTTTTTTCGTTGTCTCCAATTTAATCAAAAAATA**TTTTTT**CATTATTTACTTGATATAGCTATCTTTAAGCTGAACATTCAATATAATACATAT
	TvACT4	249200	. TAAATTTTCGGTTTTATTTGATAATTTTCTCTC**TTTTTT**AATAATTTCTCATCTTATTATATGATAACTATTGCATGATCTTTTTGTACTACTACAAATG
	TvACT5	310030	TAAACAACTTCGGTATATATTTTATAATTTATCAAC**TTTTTT**CCTTTTTTTATGAAAATTTATCTCAGATTTGTTTCCAAAGTGAAGATAATTTAAGGATAA
	TvACT6	485210	TAAATTTTCGGTCTTTATATTACTACAAATTTACTTACT**TTTTTTACTTTTT**ACCAAGCATTTCTAGAAATAATTCATTTACTTATTGGAAATCTATAAGTT
	TvACT7	149090	TAAAACAATTTCGGAATAAATATTTATTGAATTCGAAAAAAAA**TTCTTTT**AATTTCAAAAGATTTATGTTTATTAGCAAATGAAG**TTTT**AATTTCTTATATC
	TvACT8	054030	TAAAAAGTTTTTTTTTCGTTGTCTCCAATTATTCAAATC**TTTTTTTT**ATTACTGTTCGAATTCATTTAGAAGAATTGACTTTTTTTAAAGCACTATCTGGCC
	TvACT9	090470	TAAACTATATTTTATTAGCTTCCCAGTTAACAAATTTTATCAG**TTTTTT**AAATATTTTTCAATATTTTGAAATTAATCACATTCTCAACTGGCCAATTACTA
	TvACT10	337240	TAAAACAATTTGAAATGTTTCAGATTCCAAAATTTCTAA**TTTTTTT**AACTTTTTTTACTCTTTTTACAACATCTTTATCAATCTACGCCTTATATAATTCCT

The 5´- and 3´-regulatory regions of all actin-like encoding sequence were analyzed to find putative Inr, Inr-like, motif 1 (M1), motif 2 (M2), motif 3 (M3), motif 4 (M4), motif 5 (M5), polyadenylation signal (PS), cleavage site (CS) and downstream element (DSE), respectively. The manual search of these putative elements was based on reported sequences by different groups (49-53) that resulted in 10 sequences containing the necessary elements to be expressed, such as a start codon (ATG, red letter), stop codon (TAA, red letter), putative Inr/Inr-like/motifs at the 5´upstream regulatory region and putative PS, CS and DSE elements at the 3´downstream regulatory region. **Inr**, black and underlined capital letters; **motif M1**, highlighted in yellow; **motif M2**, highlighted in purple, **motif M3**, highlighted in green; **M4**, highlighted in blue; **PS**, underlined capital letters; **CS**, highlighted in gray; **DSE**, bold capital letters. See Suppl. Table S5 for more information.
