# Supplementary material for: Omics Analyses of Trichomonas vaginalis Actin and Tubulin and Their Participation in Intercellular Interactions and Cytokinesis
Source: Genes (Basel). 2022 Jun 15;13(6):1067. doi: 10.3390/genes13061067 (PMC9222396; doi:10.3390/genes13061067)
Supplement: Supplementary file 1 [file genes-13-01067-s001.zip › sentGalley Suppl. TABLES-100622/Supplementary Table S4.pdf]

Supplementary Table S4. Multiple alignment of the 5’- and 3’-regulatory sequences of the bonafide tubulin-encoding genes of *T. vaginalis*.

Group		Name	TVAG_	
**5´-regulatory regions**				+1
I	A	TvTUBα1	359090	AAAATAGAGGATGTCTGTTTGATTTTCCAAATACGTTATTAATAAAAATCCGTTTCTTTTTTCTAAAAAGTG**TCACT**CTTCATCA**ATG**
I	A	TvTUBα2	196270	AAAATAGAGGATGTCTGTTTGATTTTCCAAATACGTTATTAATAAAAATCCGTTTCTTTTTTCTAAAAAGTG**TCACT**CTTCATCA**ATG**
I	A	TvTUBα3	206890	ATAGCCATACAAACAAAATATCAAAAGACAAGTGAAAATCCGTTATGTTTTCTTGAGAAAGCAGATTTTG**ACACT**CTTCATCA**ATG**
I	A	TvTUBα4	312330	ATTTTTCATTGAAAATTAAAATACGTTAATTTTTGAATGCCGTTTTGTACGTTATCAAGAGACGGTTTTGCT**TCACT**TCATCA**ATG**
I	A	TvTUBα5	360870	CATTTATCCTTGACAATACTGTCTAAGCAAAATATCTAAAAATAGGAAAAAATTCGTTAGTAGTTTTTTAAT**TCACT**CTTCATCA**ATG**
I	A	TvTUBα6	448380	TTGCAGCAAGATTAAAAACGGATGTGTCCGTTTTTTAATAAAAAACTCTCAGAAAATATTGTAGGGTTTGT**TCAAATCAC**TCTTCATCA**ATG**
I	A	TvTUBα7	467840	TGAATTAGTAAACTGAAAACGGATTTGCCCGTTTTTTAAATAAAAACACTCAGAAAATATTGAAATGATTC**TCAAATCA**CTCTTCATCA**ATG**
I	B	TvTUBβ1	034440	TAATACGCATATTCTTTTTTATATATTTAAATTGTGATGGAGTGGTGGTTTTTTCACGTTATTCATTTTCTA**TCACTTCA**TTCTCA**ATG**
I	B	TvTUBβ2	456920	AATTACGGGACAGATAAATTATAAACTGATCTTGTGAATTATTTGAGTGAAACCGCTCAATACGTTAATA**TCATT**ATTCAC**ATG**
I	B	TvTUBβ3	008680	CACATAAATCCAGAAATAGTAAACGTGCACTTATCAAAACTATTGAGTAAAACCGCTAAATACGTTTTTAA**TCACT**ACTTTTC**ATG**
I	B	TvTUBβ4	062880	ATAAAAAGGCTCAAAAAATATAAACGCCCGGGCTGAATAAATTGAAATAAACCGAATAATTCGTTTTTAA**TCATA**ATTACAC**ATG**
**3´-regulatory regions**				
I	A	TvTUBα1	359090	……………………………………………………TAAATTCGCTAATTCCTAAAACATTATTATTAATGTTCGTAATTCGATATA**TTTTTTTT**CTCATGTTCTTTGATTTATAGTCATAATCTTA
I	A	TvTUBα2	196270	TAAATTCGCTAATTCCTAAAACATTATTATTAATGCTCATAATTAGATATA**TTTTTTTT**CTCA**TTTT**CTTAAAATTATTCAGATCCAATTT
I	A	TvTUBα3	206890	TAAATTCGCTAATTCCTAAAACATTATTATTAATGTTCGTAATTAGATATA**TTTTTT**CTCATGTTCTTTGATTTCTAGTCATAATCTTAA
I	A	TvTUBα4	312330	TAAATTCGCTAATTCCTAAAACATTATTATTAATAATATATATATA**TTTT**GATCTTTGGTTTCGATTCATAATCTTAAAATTATTCAGATC
I	A	TvTUBα5	360870	.TAAATTCGCTAATTCCTAAAAAGGTA**TTTTT**ATATCTTATGACAAACAGATTC**TTTTTTTTT**ATCTCATAATTCAAAAAGAATTTTGATA
I	A	TvTUBα6	448380	TAAGAACAATAAAAACATCTAACGTTTTCTCCTTTTAAAAAATTTTTAA**TTTTT**CATCAATAAATACTCAATTATACCTTATCATATTGACTAATCCTCCATC
I	A	TvTUBα7	467840	TAAGAAAAAAAAATTTTTTTTTAAAAATAATTC**TTTT**ATTATAATTCTCTATTCAATCTTACTCCAGTCAACTCTTGCTAATGCAGCAGCGTTTCTTACTTCA
I	B	TvTUBβ1	034440	TAAACAATTTTTATGTTGGTTCCTGATAAC**TTTTT**GTAA**TTTTTTTT**AATAAAAATTATTTCAAAATTG**TTTTT**AATTTTCATTCT
I	B	TvTUBβ2	456920	TAAGTAATCTAAACGAAGTTTCACAACATACTTTTATTTTAATTTTTTATAAGTTAC**TTTTTT**ATCATCCTTTACGAGTACATCCTTGAACGGTAA**TTTTTTTT**ATTATTCCGAAATTA
I	B	TvTUBβ3	008680	TAAGCGATTTTATATTCTGACTATAAAACATGATTTGAGTTTTGTCTTTAATCATTTCAAATTTTTACA**TTTTTTTT**CATTGTACAAATAGATTTGATAAAAAGTTTCAATTATTTTAGA
I	B	TvTUBβ4	062880	TAAGCAGTTATTTGATTTGAACGCTAAAGAATTA**TTTTTT**GGTGTAATAATTTTCTCATCCA**TTTTT**AAAAACTTCATCTATAAAAATTTGAAATTAGATATTTTGTC

The 5´- and 3´-regulatory regions of all tubulin-like encoding sequences were analyzed to find putative Inr, Inr-like, motif 1 (M1), motif 2 (M2), motif 3 (M3), motif 4 (M4), motif 5 (M5), polyadenylation signal (PS), cleavage site (CS), and downstream element (DSE), respectively. The manual search of these putative elements was based on reported sequences by different groups (49-53) that resulted in 11 sequences containing the necessary elements to be expressed, such as a start codon (ATG), stop codon (TAA/TAG), putative Inr/Inr-like/motifs at the 5´upstream regulatory region and putative PS, CS, and DSE elements at the 3´downstream regulatory region. **Inr**, black and underlined capital letters; **motif M2**, highlighted in purple, **motif M3**, highlighted in green; **motif M4**, highlighted in blue; **PS**, underlined capital letters; **CS**, highlighted in gray; **DSE**, black capital letters. See Suppl. Table S6 for more information.
