# Supplementary material for: Omics Analyses of Trichomonas vaginalis Actin and Tubulin and Their Participation in Intercellular Interactions and Cytokinesis
Source: Genes (Basel). 2022 Jun 15;13(6):1067. doi: 10.3390/genes13061067 (PMC9222396; doi:10.3390/genes13061067)
Supplement: Supplementary file 1 [file genes-13-01067-s001.zip › sentGalley Suppl. TABLES-100622/Supplementary Table S5.pdf]

Supplementary Table S5. Identification of the putative regulatory elements and position in nt at the 5´- and 3´- regulatory regions of the DNA sequences encoding for actin-like protein fragments found in the *T. vaginalis* genome database.

Group		Name	TVAG_	Inr	M1	M2	M3	M4	M5	PS	CS	DSE
		**TvACT1**	160060	In tandem TCACTTCACA (-10 to -20)				AAAAAATT (-45 to -53)		TAAA (included in the stop codon)	CAATT (32 to 37)	TTTTT (43 to 48)
		**TvACT2**	172680	In tandem TCATTTCATT (-8 to -18)			AACCGTTG (-49 to -57)			TAAA (included in the stop codon)	CAAAATT (23 to 29)	TTTTTT (36 to 42)
		**TvACT3**	200190	TCACA (-11 to 15)				GAAAAATG (-24 to -31)		TAAA (included in the stop codon)	CAATT (22 to 26)	TTTTTT (40 to 45)
		**TvACT4**	249200	TCACA (-10 to 15)	TCATTTTT (-24 to -31)					TAAA (included in the stop codon)	TAATT (19 to 23)	TTTTTT (31 to 36)
		**TvACT5**	310030	In tandem TCACTTCACA (-11 to 20)		AAAGTGAC (-73 to -80)		GAAAAATT (-45 to -53		TAAA (included in the stop codon)	TAATT (22 to 26)	TTTTTT (34 to 39)
**I**		**TvACT6**	485210	In tandem TCACTTCATT (-9 to -18)						TAAA (included in the stop codon)	CAAATT (23 to 20)	TTTTTT (37 to 42)
		**TvACT7**	149090	TCACTTCACA (-11 to -20)			AACCGTTT (-73 to -80)			TAAA (included in the stop codon)<br>TAAA (14 to 17)	GAATT (26 to 30)<br>TAATT (47 to 51)	TTTT (44 to 47)<br>TTTT (83 to 86)
		**TvACT8**	054030	TCACATCACA (-11 to -20)		AAAGTGAC (-73 to -80)				TAAA (included in the stop codon)	CAATT (23 to 27)	TTTTTTTT (37 to 44)
		**TvACT9**	090470	TCACA (-11 to -16)						TAAA (included in the stop codon)	CAAATT (28 to 33)	TTTTTT (41 to 46)
		**TvACT10**	337240	TCATT (-10 to -14)		AAAGTGAC (-16 to -23)				TAAA (included in the stop codon)	CAAAATT (25 to 31)	TTTTTTT (37 to 43)
			512800				AACGGCTC (-5 to -12)					
	**A**		150270	TCATT (-9 to -13)		AACCGCTG (-49 to -56)				TAAA (included in the stop codon)		
**II**			534990							TAAA (included in the stop codon)	GAAATT (30 to 35)	TTTTTTTT (41 to 48)
			371880	TCATT (-14 to -18)			AACCGTTT (-82 to -89)			TAAA (included in the stop codon)	TAAATT (28 to 33)	TTTTT (62 to 66)
			043970	TCATA (-4 to -8)						TAAA (included in the stop codon)	TAAATT (11 to 16)	
			094140					AAAAAATT (-18 to -25)		TAAA (included in the stop codon)	TAAAATT (64 to 70)	TTTTT (86 to 90)

B	189860	TCAGA (-12 to -16)	TCATTTTC (-61 to- 68)			TAAA (included in the stop codon)<br>TAAA (17 to 20 nt)	CAAAAT (28 to 33)<br>TAAATT (53 to 58)	TTTTTTT (43 to 49)<br>TTTTTT (75 to 80)
	027630	TCAAA (-51 to -55)				TAAA (31 to 34)	GAATT (58 to 62)	TTTTT (90 to 94)
	161530	CCAAA (-11 to -15)				TAAA (included in the stop codon)	TAAAATTA (40 to 47)	TTTT (71 to 74)
	434970	TCATT (-4 to -8)			AAAAAATT (-8 to -15)	TAAA (6 to 9)	TAATT (45 to 49)	
C	248040	CCAAA (-5 to -9)				TAAA (44 to 47)	TAATT (62 to 66)	TTTTT (68 to 72)
	071770				TAAAAAAAG (-1 to -9)	TAAA (included in the stop codon)	TAATT (24 to 28)	TTTTTT (55 to 60)
	495500			AAAGTGAC (-2 to -9)		TAAA (24 to 27)	TAATT (35 to 39)	TTTTT (75 to 78)
	247170	TCAAG (-18 to -22)				TAAA (39 to 42)	TAATT (52 to 56)	TTTTT (83 to 87)
	167330	TCATA (-27 to -31)				TAAA (14 to 17)	TAAATT (41 to 46)	TTTTT (50 to 54)
	276270	TCAAA (-13 to -17)	TCACTTTT (-44 to -51)	AAAGTGAC (-64 to -71)		TAAA (included in the stop codon)	TAAAAATT (33 to 40)	TTTTTT (58 to 63)
	225210	TCATT (-10 to -14)		AAAGTGAT (-67 to -74)		TAAA (included in the stop codon)	GAAATT (50 to 54)	
	354260	TCATA (-10 to -14)			AAAAAATT (-42 to -49)	TAAA (34 to 37)		TTTTTT (63 to 68)
	238100	TCATA (-35 to -39)				TAAA (40 to 43)	TAAATT (62 to 67)	TTTT (86 to 89)

The 5´- and 3´-regulatory regions of all sequences encoding for actin-like proteins were downloaded from each contig including 100 bp up and downstream of each ORF found in the *Tv* genome database, analyzed to find putative promoter like elements, such as Inr, Inr-like, motif 1 (M1), motif 2 (M2), motif 3 (M3), motif 4 (M4), motif 5 (M5), or polyadenylation signal (PS), cleavage site (CS), and downstream element (DSE), respectively. We did a manual search of these putative elements to identify them in all DNA sequences. The search was based on reported sequences by different groups (49-53; *extra ref a-l). Several DNA sequences contain the necessary elements to be expressed, such as a start codon (ATG), stop codon (TAA/TAG), and putative promoter-like motifs in the 5´-upstream regulatory region and putative PS, CS, and DSE elements in the 3´-downstream regulatory regions. Some other sequences only possess regulatory elements in the 5'- upstream regulatory regions or only regulatory elements in the 3´- downstream regulatory regions. However, we did not find regulatory elements for some sequences either at the 5'- or 3'- end.

***Extra references with 5'- and 3'-regulatory sequences in different reported genes.**

a) Figueroa-Angulo E. E., Estrella-Hernández P., Salgado-Lugo H., Ochoa-Leyva A., Gómez Puyou A., Campos S. S., Montero-Moran G., Ortega-López J., Saab-Rincón G. and Arroyo R. 2012. Cellular and biochemical characterization of two closely related triosephosphate isomerases from *Trichomonas vaginalis*. Parasitology. 139(13):1729 – 1738.

b) Hernández-Romano P., Hernández R., Arroyo R., Alderete J. and López-Villaseñor I. 2010. Identification and characterization of a surface-associated, subtilisin-like serine protease in *Trichomonas vaginalis*. Parasitology, 137(11):1621-1635.

c) León-Félix J., Ortega-López J., Orozco-Solís R. and Arroyo R. 2004. Two novel asparaginyl endopeptidase-like cysteine proteinases from the protist *Trichomonas vaginalis*: their evolutionary relationship within the clan CD cysteine proteinases. Gene. 335:25-35.

d) León-Sicairos C. R., León-Félix J. and Arroyo R. 2004. tvcp12: a novel *Trichomonas vaginalis* cathepsin L-like cysteine proteinase-encoding gene. Microbiology. 150(5):1131-1138.

e) Liston D. R. and Johnson P. J. 1999. Analysis of a ubiquitous promoter element in a primitive Eukaryote: early evolution of the initiator element. Molecular and Cellular Biology. 19, 2380-2388.

f) Mancilla-Olea M. I., Ortega-López J., Figueroa-Angulo E. E., Avila-González L., Cárdenas-Guerra R. E, Miranda-Ozuna J. F. T., González-Robles A., Hernández-García M. S., Sánchez-Ayala L. and Arroyo R. 2018. *Trichomonas vaginalis* cathepsin D-like aspartic proteinase (Tv-CatD) is positively regulated by glucose and degrades human hemoglobin. The International Journal of Biochemistry & Cell Biology. Volume 97:1-15.

g) Puente-Rivera J., Ramón-Luing L. A., Figueroa-Angulo E. E., Ortega-López J. and Arroyo R. 2014. Trichocystatin-2 (TC-2): An endogenous inhibitor of cysteine proteinases in *Trichomonas vaginalis* is associated with TvCP39. The International Journal of Biochemistry & Cell Biology. 54:255-265.

h) Puente-Rivera J., Villalpando J. L., Villalobos-Osnaya A., Vázquez-Carrillo L. I., León-Ávila G., Ponce-Regalado M. D., López-Camarillo C., Elizalde-Contreras J. M., Ruiz-May E., Arroyo R., Alvarez-Sánchez M. E. 2017. The 50kDa metalloproteinase TvMP50 is a zinc-mediated *Trichomonas vaginalis* virulence factor. Molecular and Biochemical Parasitology. 217:32-41.

i) Ramón-Luing L. A., Rendón-Gandarilla F. J., Puente-Rivera J., Ávila-González L. and Arroyo R. 2011. Identification and characterization of the immunogenic cytotoxic TvCP39 proteinase gene of *Trichomonas vaginalis*. The International Journal of Biochemistry & Cell Biology. 43(10):1500-1511.

j) Smith A. J., Chudnovsky L., Simoes-Barbosa A., Delgadillo-Correa M. G., Jonsson Z. O., Wohlschlegel J. A., and Johnson P. J. 2011. Novel core promoter elements and a cognate transcription factor in the divergent unicellular eukaryote *Trichomonas vaginalis*. Molecular and cellular biology, 31(7), 1444–1458.

k) Solano-González E., Burrola-Barraza E., León-Sicairos C., Avila-González L., Gutiérrez-Escolano L., Ortega-López J. and Arroyo, R. 2007. The trichomonad cysteine proteinase TVCP4 transcript contains an iron-responsive element. *FEBS Letters*. 581.

l) Amos B., Aurrecoechea C., Barba M, Barreto A, Basenko E. Y., Bażant W., Belnap R., Blevins A. S., Böhme U., Brestelli J., Brunk B. P., Caddick M., Callan D., Campbell L., Christensen M. B., Christophides G. K., Crouch K., Davis K., DeBarry J., Doherty R., Duan Y., Dunn M., Falke D., Fisher S., Flicek P., Fox B., Gajria B., Giraldo-Calderón G. I., Harb O. S., Harper E., Hertz-Fowler C., Hickman M. J., Howington C., Hu S., Humphrey S., Iodice J., Jones A., Judkins J., Kelly S. A., Kissinger J. C., Kun Kwon D., Lamoureux K, Lawson D., Li W., Lies K., Lodha D., Long J., MacCallum R. M., Maslen G., McDowell M. A., Nabrzyski J., Roos D. S., Rund S. S. C., Wever Schulman S., Shanmugasundram A., Sitnik V., Spruill D., Starns D., Stoeckert C. J., Tomko S. S., Wang H, Warrenfeltz S., Wieck R., Wilkinson P. A., Xu L. and Zheng J. 2022. VEuPathDB: the eukaryotic pathogen, vector and host bioinformatics resource center. *Nucleic Acids Research*. 50(D1):D898–D911.
