# Supplementary material for: Omics Analyses of Trichomonas vaginalis Actin and Tubulin and Their Participation in Intercellular Interactions and Cytokinesis
Source: Genes (Basel). 2022 Jun 15;13(6):1067. doi: 10.3390/genes13061067 (PMC9222396; doi:10.3390/genes13061067)
Supplement: Supplementary file 1 [file genes-13-01067-s001.zip › sentGalley Suppl. TABLES-100622/Supplementary Table S6.pdf]

Supplementary Table S6. Identification of the putative regulatory elements and position at the in 5´- and 3´- regulatory regions of the DNA sequences encoding for tubulin-like proteins found in the *T. vaginalis* genome database.

Group		Name	TVAG_	Inr	M1	M2	M3	M4	M5	PS	CS	DSE
		**TvTUBα1**	359090	TCACT<br>(-9 to -13)		AAAGTGTC<br>(-12 to -19)		TAAAAATC<br>(-36 to -44)		TAAA<br>(included in the stop codon)<br>TAAA<br>(14 to 17)	TAATT<br>(7 to 11)<br>TAATT<br>(37 to 41)	TTTTTTT<br>(49 to 55)
		**TvTUBα2**	196270	TCACT<br>(-9 to -13)		AAAGTGTC<br>(-12 to -19)		TAAAAATC<br>(-36 to -44)		TAAA<br>(included in the stop codon)<br>TAAA<br>(14 to 17)	TAATT<br>(7 to 11)<br>TAATT<br>(37 to 41)	TTTTTTT<br>(49 to 55)<br>TTTT<br>(60 to 63)
	**A**	**TvTUBα3**	206890	ACACT<br>(-9 to -13)						TAAA<br>(included in the stop codon)<br>TAAA<br>(14 to 17)	TAATT<br>(7 to 11)<br>TAATT<br>(37 to 41)	TTTTT<br>(49 to 54)
		**TvTUBα4**	312330	TCACT<br>(-7 to -11)			GACGGTTT<br>(-16 to -23)			TAAA<br>(included in the stop codon)	TAATT<br>(7 to 11)	TTTT<br>(42 to 45)
**I**		**TvTUBα5**	360870	TCACT<br>(-9 to -11)				TAAAAATA<br>(-42 to -49)<br>AAAAAATT<br>(-32 to -39)		TAAA<br>(included in the stop codon)<br>TAAA<br>(14 to 17)	TAATT<br>(7 to 11)	TTTTT<br>(24 to 28)<br>TTTTTTTT<br>(51 to 58)
		**TvTUBα6**	448380	TCAAATCAC<br>(-10 to -18)						TAAA<br>(7 to 10)	AAATT<br>(37 to 41)	TTTTT<br>(47 to 51)
		**TvTUBα7**	467840	TCAAATCA<br>(-11 to -18)						TAAA<br>(19 to 22)	TAATT<br>(25 to 29)	TTTT<br>(31 to 34)
	**B**	**TvTUBβ1**	034440	TCACTTCA<br>(-7 to -14)						TAAA<br>(included in the stop codon)<br>TAAA<br>(46 to 49)	CAATT<br>(2 to 6)<br>AAATT<br>(60 to 64)<br>TAATT<br>(70 to 74)	TTTTT<br>(28 to 32)<br>TTTTTTT<br>(37 to 43)<br>TTTTT<br>(66 to 70)
		**TvTUBβ2**	456920	TCATT<br>(-7 to -11)						TAAA<br>(7 to 10)	CAATT<br>(37 to 41)	TTTTTT<br>(55 to 60)<br>TTTTTTT<br>(94 to 100)
		**TvTUBβ3**	008680	TCACT<br>(-8 to -12)						TAAA<br>(21 to 24)	AAATT<br>(56 to 60)	TTTTTTT<br>(67 to 73)
		**TvTUBβ4**	062880	TCATA<br>(-8 to -12)				AAAAAATA<br>(-63 to -70)		TAAA<br>(22 to 25)	AATT<br>(27 to 30)<br>TAATT<br>(45 to 49)	TTTTTT<br>(32 to 37)<br>TTTTT<br>(60 to 64)
	**A**		109820	TCATT<br>(-15 to -19)	TCATTTTT<br>(-12 to -19)<br>TCACTTTT<br>(-26 to -33)			AAAAAATA<br>(-54 to -61)		TAAA<br>(included in the stop codon)<br>TAAA<br>(11 to 14)<br>TAAA<br>(15 to 18)	TAATT<br>(78 to 82)	
			338530	TCATA<br>(-8 to -12)	CAAAATTT<br>(-38 to 45)					TAAA<br>(24 to 27)	TAATT<br>(32 to 36)<br>AAATT<br>(46 to 50)	TTTTT<br>(59 to 63)<br>TTTTT<br>(69 to 73)
			207590	TCAAA<br>(-7 to -11)				TAAAAATT<br>(-28 to -35)<br>GAAAAATT<br>(-17 to -24)		TAAA<br>(1 to 4)	TAATT<br>(11 to 15)<br>TAATT<br>(23 to 27)<br>AAATTT<br>(33 to 38)	

II	B		525430	Without regulatory regions								
			523980	Without regulatory regions and ORF								
			519620	Without regulatory regions								
			073810	TCACA (-8 to -12)								
			289290	ACAGT (-20 to -24)						TAAA (24 to 27)	TAATT (32 to 36) AAATT (46 to 50)	TTTTT (59 to 63) TTTT (67 to 70)
			200200							TAAA (18 to 21) TAAA (27 to 30)	TATT (53 to 57) AATT (72 to 76) AATT (78 to 81)	
			024080	Without regulatory regions and ORF								
			345420					AAAAAACG (-31 to -38)		TAAA (included in the stop codon)	AAATT (9 to 12)	
			148390	TCACA (-8 to -12)								
			148400	TCACA (-25 to -29)						TAAA (included in the stop codon) TAAA (4 to 7)	TAATT (44 to 48)	TTTT (83 to 86)
			043330	TCACT (-17 to -21)						TAAA (79 to 82)		TTTTTTT (94 to 100)
			448410	TCACT (-17 to -21)						TAAA (51 to 54) TAAA (61 to 64)		TTTT (66 to 69) TTTTTTT (71 to 77)
	C		065740	TCACT (-2 to -6)	TCATTTTT (-14 to -21)					TAAA (38 to 41)		
			184510	TAATT (Inr-like) (-15 to -19)						TAAA (included in the stop codon) TAAA (15 to 18)	AATT (44 to 47)	TTTTTTTT (36 to 43)
			369500	TCATT (-15 to -19)	TCACTTTT (-26 to -33) TCATTTTT (-12 to -19)			AAAAAATA (-54 to -61)		TAAA (29 to 32)	AAATT (79 to 83)	
			073800	TCACA (-25 to -29)						TAAA (51 to 54)	AAATT (60 to 64)	
			257730	TCACT (-17 to -21)								

The 5´- and 3´-regulatory regions of all DNA sequence encoding for tubulin-like proteins were downloaded from each contig including 100 bp up and downstream of each ORF found in the *Tv* genome database, analyzed to find putative promoter elements or motifs Inr, Inr-like, motif 1 (M1), motif 2 (M2), motif 3 (M3), motif 4 (M4), motif 5 (M5), or/and polyadenylation signal (PS), cleavage site (CS) and downstream element (DSE), respectively. We did a manual search of these putative elements to find them in all sequences. The search was based on reported sequences by different groups (49-53; *extra ref a-l). Several sequences contain the necessary elements to be expressed, since they possess a start codon (ATG), stop codon (TAA/TAG), putative Inr/Inr-like/motifs in the 5´-upstream regulatory region and putative PS, CS, and DSE elements in the 3´-downstream regulatory region. Others only possess regulatory elements in the 5'- upstream regulatory regions or only regulatory elements in the 3´-downstream regulatory regions. We did not find regulatory sequences in four DNA sequences and two of them did not have an open reading frame (ORF), and lack of the ATG star codon.

***Extra references with 5'- and 3'-regulatory sequences in different reported genes.**

a) Figueroa-Angulo E. E., Estrella-Hernández P., Salgado-Lugo H., Ochoa-Leyva A., Gómez Puyou A., Campos S. S., Montero-Moran G., Ortega-López J., Saab-Rincón G. and Arroyo R. 2012. Cellular and biochemical characterization of two closely related triosephosphate isomerases from *Trichomonas vaginalis*. Parasitology. 139(13):1729 – 1738.

b) Hernández-Romano P., Hernández R., Arroyo R., Alderete J. and López-Villaseñor I. 2010. Identification and characterization of a surface-associated, subtilisin-like serine protease in *Trichomonas vaginalis*. Parasitology, 137(11):1621-1635.

c) León-Félix J., Ortega-López J., Orozco-Solís R. and Arroyo R. 2004. Two novel asparaginyl endopeptidase-like cysteine proteinases from the protist *Trichomonas vaginalis*: their evolutionary relationship within the clan CD cysteine proteinases. Gene. 335:25-35.

d) León-Sicairos C. R., León-Félix J. and Arroyo R. 2004. tvcp12: a novel *Trichomonas vaginalis* cathepsin L-like cysteine proteinase-encoding gene. Microbiology. 150(5):1131-1138.

e) Liston D. R. and Johnson P. J. 1999. Analysis of a ubiquitous promoter element in a primitive Eukaryote: early evolution of the initiator element. Molecular and Cellular Biology. 19, 2380-2388.

f) Mancilla-Olea M. I., Ortega-López J., Figueroa-Angulo E. E., Avila-González L., Cárdenas-Guerra R. E, Miranda-Ozuna J. F. T., González-Robles A., Hernández-García M. S., Sánchez-Ayala L. and Arroyo R. 2018. *Trichomonas vaginalis* cathepsin D-like aspartic proteinase (Tv-CatD) is positively regulated by glucose and degrades human hemoglobin. The International Journal of Biochemistry & Cell Biology. Volume 97:1-15.

g) Puente-Rivera J., Ramón-Luing L. A., Figueroa-Angulo E. E., Ortega-López J. and Arroyo R. 2014. Trichocystatin-2 (TC-2): An endogenous inhibitor of cysteine proteinases in *Trichomonas vaginalis* is associated with TvCP39. The International Journal of Biochemistry & Cell Biology. 54:255-265.

h) Puente-Rivera J., Villalpando J. L., Villalobos-Osnaya A., Vázquez-Carrillo L. I., León-Ávila G., Ponce-Regalado M. D., López-Camarillo C., Elizalde-Contreras J. M., Ruiz-May E., Arroyo R., Alvarez-Sánchez M. E. 2017. The 50kDa metalloproteinase TvMP50 is a zinc-mediated *Trichomonas vaginalis* virulence factor. Molecular and Biochemical Parasitology. 217:32-41.

i) Ramón-Luing L. A., Rendón-Gandarilla F. J., Puente-Rivera J., Ávila-González L. and Arroyo R. 2011. Identification and characterization of the immunogenic cytotoxic TvCP39 proteinase gene of *Trichomonas vaginalis*. The International Journal of Biochemistry & Cell Biology. 43(10):1500-1511..j) Smith A. J., Chudnovsky L., Simoes-Barbosa A., Delgadillo-Correa M. G., Jonsson Z. O., Wohlschlegel J. A., and Johnson P. J. 2011. Novel core promoter elements and a cognate transcription factor in the divergent unicellular eukaryote *Trichomonas vaginalis*. Molecular and cellular biology, 31(7), 1444–1458.

k) Solano-González E., Burrola-Barraza E., León-Sicairos C., Avila-González L., Gutiérrez-Escolano L., Ortega-López J. and Arroyo, R. 2007. The trichomonad cysteine proteinase TVCP4 transcript contains an iron-responsive element. *FEBS Letters*. 581.

l) Amos B., Aurrecoechea C., Barba M, Barreto A, Basenko E. Y., Bażant W., Belnap R., Blevins A. S., Böhme U., Brestelli J., Brunk B. P., Caddick M., Callan D., Campbell L., Christensen M. B., Christophides G. K., Crouch K., Davis K., DeBarry J., Doherty R., Duan Y., Dunn M., Falke D., Fisher S., Flicek P., Fox B., Gajria B., Giraldo-Calderón G. I., Harb O. S., Harper E., Hertz-Fowler C., Hickman M. J., Howington C., Hu S., Humphrey S., Iodice J., Jones A., Judkins J., Kelly S. A., Kissinger J. C., Kun Kwon D., Lamoureux K, Lawson D., Li W., Lies K., Lodha D., Long J., MacCallum R. M., Maslen G., McDowell M. A., Nabrzyski J., Roos D. S., Rund S. S. C., Wever Schulman S., Shanmugasundram A., Sitnik V., Spruill D., Starns D., Stoeckert C. J., Tomko S. S., Wang H, Warrenfeltz S., Wieck R., Wilkinson P. A., Xu L. and Zheng J. 2022. VEuPathDB: the eukaryotic pathogen, vector and host bioinformatics resource center. *Nucleic Acids Research*. 50(D1):D898–D911.
