# Supplementary material for: Omics Analyses of Trichomonas vaginalis Actin and Tubulin and Their Participation in Intercellular Interactions and Cytokinesis
Source: Genes (Basel). 2022 Jun 15;13(6):1067. doi: 10.3390/genes13061067 (PMC9222396; doi:10.3390/genes13061067)
Supplement: Supplementary file 1 [file genes-13-01067-s001.zip › sentGalley Suppl. TABLES-100622/Supplementary Table S7.pdf]

Supplementary Table S7. Protein expression of the actin-like encoding gene fragments in proteomes reported of different *T. vaginalis* isolates and under distinct culture conditions.

Isolate			CNCD147[23,27]		T1[19,21]		FMV1[15]		1[28]	FF28jT-Rio[16]	SD7[18]	B7268[18]	G3[18]	SD10[18]	LSU 160[29]	Unknown[20]	B7RC2[24,26]		RU393[25]	TV17-48[30]	FMV1[17]	FF28jT-Rio[17]	FMV1[22]		
Condition/Morph			Put	Zn^{2+}	IR	HI	TF	CLS	TF	IR	HI					Am	Ca^{2+}	Suc	HI/Am	Unknown	HVir	LVir	Infection (min)		
Extract/Organelle			TE	TE	Hyd	Hyd	TE	TE	TE	TE	MEP					TE	MVs	Exo	MEP	Lys	TE	TE	5	30	120
Group	Name	TVAG_																							
I	TvACT1	160060					xx	x		x						±	x						x	x	x
I	TvACT2	172680					xx	x		x						±	x						x	x	x
I	TvACT3	200190	x		xx		xx	x		x							x							x	x
I	TvACT4	249200			±		xx	x		x							x						x		x
I	TvACT5	310030					xx	x		x							x				xx	x	x	x	x
I	TvACT6	485210					xx	x		x							x						x	x	x
I	TvACT7	149090				x	xx	x		x							x						x		x
I	TvACT8	54030	x	x			xx	x			x	x	x	x	x	±	x		x				x	x	x
I	TvACT9	90470					xx	x							x		x	x	x				x	x	x
I	TvACT10	337240		x			xx	x							x		x	x	x	x			x		
II A		512800													x		x		x				x	x	x
II A		150270													x				x					x	x
II A		534990													x		x		x				x	x	x
II B		371880			xx		x	xx	x						x		x	x	x	x	xx	x			
II B		043970					xx	x																	
II B		094140					x	xx												x					
II B		189860					xx	x																	
II B		027630					x	xx																	
II B		161530																		x					
II B		434970			±		xx	x	x									x	x	x					
II C		248040																							
II C		071770																							
II C		495500																							
II C		247170																							
II C		167330																							
II C		276270																							
II C		225210																							
II C		354260																							
II C		238100																							

Morph: Morphology; TE: Total Extract; Put: Putrescine; Zn^{2+}: Zinc; IR: Iron-restriction; HI: High iron; Ca^{2+}: Calcium; Suc: Sucrose; Hyd: Hydrogenosomes; TF: Trophozoite; CLS: Cyst-Like Structure; MEP: Membrane-Enriched Pellet; Am: Amoeboid; HVir: High virulence; LVir: Low virulence; MVs Microvesicles; Exo: Exosome; Lys: Lysosome; X: Presence; XX: Overexpressed; ±: Down expression. Superindex numbers of references: [15]Dias-Lopes et al., 2018; [16]de Jesus et al., 2007; [17]Cuervo et al., 2008; [18]de Miguel et al., 2010; [19]Schneider et al., 2011; [20]Huang et al., 2012; [21]Beltrán et al., 2013; [22]Gould et al., 2013; [23]Quintas-Granados et al., 2013; [24]Twu et al., 2013; [25]Riestra et al., 2015; [26]Nievas et al., 2017; [27]Alvarez-Sánchez et al., 2018; [28]Beri et al., 2020; [29]Molgora et al., 2021; [30]Zimmann et al., 2022.
