# Supplementary material for: Omics Analyses of Trichomonas vaginalis Actin and Tubulin and Their Participation in Intercellular Interactions and Cytokinesis
Source: Genes (Basel). 2022 Jun 15;13(6):1067. doi: 10.3390/genes13061067 (PMC9222396; doi:10.3390/genes13061067)
Supplement: Supplementary file 1 [file genes-13-01067-s001.zip › sentGalley Suppl. TABLES-100622/Supplementary Table S8.pdf]

Supplementary Table S8. Protein expression of tubulin-like encoding gene fragments in proteomes reported of different *T. vaginalis* isolates and under distinct culture conditions.

Isolate				T1[19, 21]		FMV1[15]		1[28]		PA[18]	SD7[18]	B7268[18]	G3[18]	SD10[18]	T1[18]	LSU 160[29]	Unknown[20]	B7RC2[24,26]		RU393[25]	TV17-48[30]	FMV1[22]		
Condition/Morphology				IR	HI	TF	CLS	TF	CLS	HI							Am	Ca^{2+}	Suc	HI/Am	Uknown	Infection time (min)		
Extract/Organelle				Hyd	Hyd	TE	TE	TE	TE	MEP							TE	MVs	Exo	MEP	Lys	5	30	120
Group		Name	TVAG_																					
I	A	TvTUBα1	359090			x	xx											x				x		
		TvTUBα2	196270			x	xx	x	x	x	x	x	x	x	x		±	x		x		x	x	x
		TvTUBα3	206890			x	xx											x				x	x	x
		TvTUBα4	312330			x	xx											x						
		TvTUBα5	360870	±		x	xx											x						
		TvTUBα6	448390	±										x	x					x				
		TvTUBα7	467840																					
	B	TvTUBβ1	34440	xx		x		x								x		x						
		TvTUBβ2	456920	±		x	xx											x	x			x		
		TvTUBβ3	008680		x	x	xx				x	x	x		x			x	x	x		x		x
		TvTUBβ4	062880			x	xx											x	x			x	x	x
II	A		109820			xx	xx														x			
			338530													x					x			
			207590																					
	B		525430					x								x		x			x			
			523980													x								
			519620													x								
			073810													x								
			289290	±												x								
			200200													x								
			024080													x						x	x	x
			345420													x								
			148390													x	±							
			148400																			x		x
			043330													x								
			448410													x								
	C		065740																					
			184510																					
			369500																					
			073800																					
			257730																					

Morph: Morphology; TE: Total Extract; IR: Iron-restriction; HI: High iron; Ca^{2+}: Calcium; Suc: Sucrose; Hyd: Hydrogenosomes; TF: Trophozoite; CLS: Cyst-Like Structure; MEP: Membrane-Enriched Pellet; Am: Amoeboid; MVs Microvesicles; Exo: Exosome; Lys: Lysosome; X: Presence; XX: Overexpressed; ±: Down expression. Superindex numbers of references: [15]Dias-Lopes et al., 2018; [18]de Miguel et al., 2010; [19]Schneider et al., 2011; [20]Huang et al., 2012; [21]Beltrán et al., 2013; [22]Gould et al., 2013; [24]Twu et al., 2013; [25]Riestra et al., 2015; [26]Nievas et al. 2017; [28]Beri et al., 2020; [29]Molgora et al., 2021; [30]Zimmann et al., 2022.
