# Supplementary material for: Omics Analyses of Trichomonas vaginalis Actin and Tubulin and Their Participation in Intercellular Interactions and Cytokinesis
Source: Genes (Basel). 2022 Jun 15;13(6):1067. doi: 10.3390/genes13061067 (PMC9222396; doi:10.3390/genes13061067)
Supplement: Supplementary file 1 [file genes-13-01067-s001.zip › sentGalley Suppl. TABLES-100622/Supplementary Table S9[13049].pdf]

Supplementary Table S9. Predicted 3D structures of actin-like aa deduced sequences found in the *T. vaginalis* genome database.

Protein			Template	Sequence Identity (%)	Description	GMQE	Ramachandran Favoured (%)	3D Model
Group	Name	TVAG_						
I	TvACT1	160060	3ci5.1.A	75.94	Major actin complex of phosphorylated *Dictyostelium discoideum* actin with gelsolin	0.85	97.84	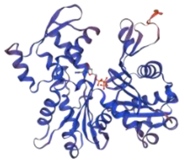
	TvACT2	172680	3ci5.1.A	75.94	Major actin complex of phosphorylated *Dictyostelium discoideum* actin with gelsolin	0.86	97.84	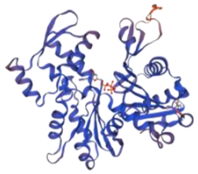
	TvACT3	200190	3ci5.1.A	75.94	Major actin complex of phosphorylated *Dictyostelium discoideum* actin with gelsolin	0.84	97.84	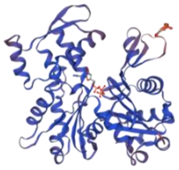
	TvACT4	249200	3ci5.1.A	75.94	Major actin complex of phosphorylated *Dictyostelium discoideum* actin with gelsolin	0.85	97.84	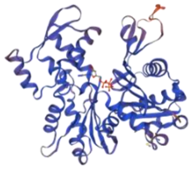
	TvACT5	310030	3ci5.1.A	75.94	Major actin complex of phosphorylated *Dictyostelium discoideum* actin with gelsolin	0.85	97.84	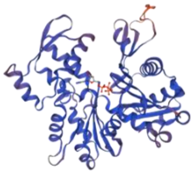
	TvACT6	485210	3ci5.1.A	75.94	Major actin complex of phosphorylated *Dictyostelium discoideum* actin with gelsolin	0.85	97.84	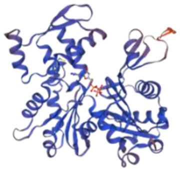
	TvACT7	149090	3ci5.1.A	75.94	Major actin complex of phosphorylated *Dictyostelium discoideum* actin with gelsolin	0.85	97.84	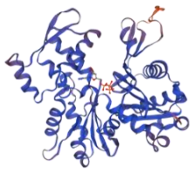

		TvACT8	54030	3ci5.1.A	75.67	Major actin complex of phosphorylated *Dictyostelium discoideum* actin with gelsolin	0.85	97.84	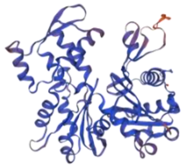
		TvACT9	90470	3ci5.1.A	75.94	Major actin complex of phosphorylated *Dictyostelium discoideum* actin with gelsolin	0.85	97.84	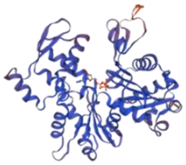
		TvACT10	337240	3ci5.1.A	75.94	major actin complex of phosphorylated *dictyostelium discoideum* actin with gelsolin	0.85	97.84	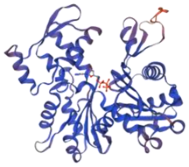
			512800	2asm.1.A	75.14	Actin, alpha skeletal muscle structure of rabbit actin in complex with *Reidispongiolide* A	0.86	98.35	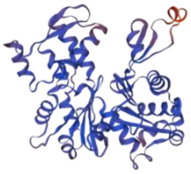
	**A**		150270	3ci5.1.A	75.36	Major actin complex of phosphorylated *Dictyostelium discoideum* actin with gelsolin	0.85	97.95	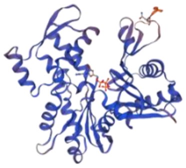
			534990	4b1w.1.A	76.05	Actin, alpha skeletal muscle structure of the Phactr1 RPEL-2 domain bound to actin	0.87	97.45	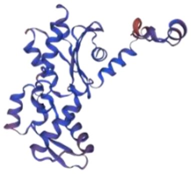
			371880	4jd2.1.A	52.93	Actin-related protein 3 crystal structure of *Bos taurus* Arp2/3 complex binding with *Mus musculus* GMF	0.80	94.90	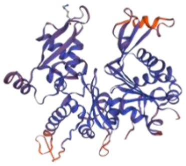
			043970	5i9e.1.A	23.04	Actin-related protein 4 crystal structure of a nuclear actin ternary complex	0.59	90.05	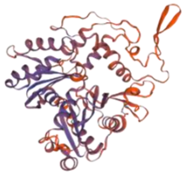

			094140	4efh.1.A	43.16	Actin-1 *Acanthamoeba* actin complex with Spir domain D	0.74	97.01	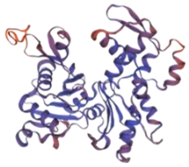
	**B**		189860	7q8b.1.A	25.22	Actin *Leishmania major* actin filament in ADP-Pi state	0.62	92.11	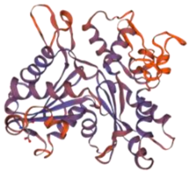
			027630	2asm.1.A	46.70	Actin, alpha skeletal muscle structure of rabbit actin in complex with *Reidispongiolide* A	0.80	97.21	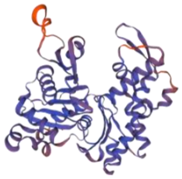
**II**			161530	2asm.1.A	38.55	Actin, alpha skeletal muscle structure of rabbit actin in complex with *Reidispongiolide* A	0.75	95.74	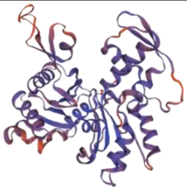
			434970	1nmd.1.A	26.85	Actin crystal structure of *D. discoideum* actin-gelsolin segment 1 complex crystallized in the presence of Lithium ATP	0.71	95.00	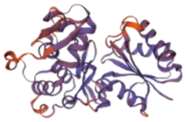
			248040	1yvn.1.A	39.37	Protein (actin) the yeast actin Val 159 Asn mutant complex with human gelsolin segment 1.	0.44	94.60	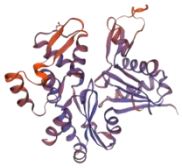
			071770	5i9e.1.A	21.99	Actin-related protein 4 crystal structure of a nuclear actin ternary complex	0.60	91.83	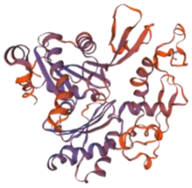
			495500	5i9e.1.A	19.26	Actin-related protein 4 crystal structure of a nuclear actin ternary complex	0.59	91.71	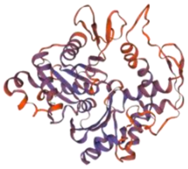

			247170	3ci5.1.A	49.87	Major actin complex of phosphorylated *Dictyostelium discoideum* actin with gelsolin	0.79	97.83	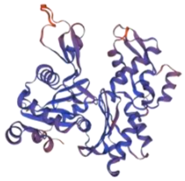
			167330	4cbw.1.A	33.61	Actin, alpha skeletal muscle, actin crystal structure of *Plasmodium berghei* actin I with D-loop from muscle actin	0.72	92.58	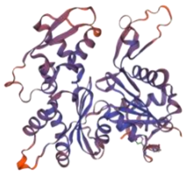
	**C**		276270	3ci5.1.A	43.63	Major actin complex of phosphorylated *Dictyostelium discoideum* actin with gelsolin	0.78	95.90	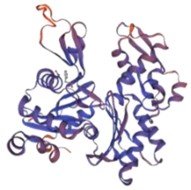
			225210	3ci5.1.A	34.73	Major actin complex of phosphorylated *Dictyostelium discoideum* actin with gelsolin	0.76	95.49	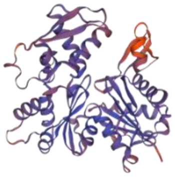
			354260	3ci5.1.A	23.69	Major actin complex of phosphorylated *Dictyostelium discoideum* actin with gelsolin	0.64	94.46	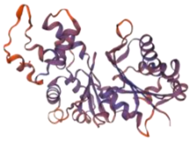
			238100	4cbu.1.A	58.77	Actin-1 crystal structure of *Plasmodium falciparum* actin I	0.71	96.33	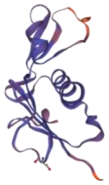

3D Structure prediction for each deduced actin-like protein fragments of the *Tv* genome database was obtained in the Swiss Model Server from EXPASY (https://swissmodel.expasy.org/) [Waterhouse, A., Bertoni, M., Bienert, S., Studer, G., Tauriello, G., Gumienny, R., Heer, F.T., de Beer, T.A.P., Rempfer, C., Bordoli, L., Lepore, R., and Schwede, T. 2018. SWISS-MODEL: homology modelling of protein structures and complexes. Nucleic Acids Res. 46, W296-W303; Xu J, and Zhang Y. 2010. How significant is a protein structure similarity with TM-score = 0.5? Bioinformatics. 26(7):889-95] using the deduced aa sequence. These data were one of the parameters to consider for the identification of possible bonafide actin proteins (Group I) and actin-like protein segments (Group II) in *Tv*. **GMQE**, Global Model Quality Estimate. It is a quality estimate, which combines properties from the target-template alignment and the template 3D structure. GMQE gives an overall 3D model quality measurement between 0 and 1, the higher numbers indicating higher expected quality (Waterhouse *et al*., 2018).
